# Analysis of tetra- and hepta-nucleotides motifs promoting -1 ribosomal frameshifting in *Escherichia coli*

**DOI:** 10.1093/nar/gku386

**Published:** 2014-05-28

**Authors:** Virag Sharma, Marie-Françoise Prère, Isabelle Canal, Andrew E. Firth, John F. Atkins, Pavel V. Baranov, Olivier Fayet

**Affiliations:** 1 School of Biochemistry and Cell biology, University College Cork, Cork, Ireland; 2 Laboratoire de Microbiologie et Génétique moléculaire, UMR5100, Centre National de la Recherche Scientifique, Université Paul Sabatier-Toulouse III, 118 route de Narbonne, Toulouse 31062-cedex, France; 3 Department of Pathology, University of Cambridge, Cambridge CB2 1QP, UK; 4 Department of Human Genetics, University of Utah, 15N 2030E, Rm7410, Salt Lake City, UT 84112-5330, USA

## Abstract

Programmed ribosomal -1 frameshifting is a non-standard decoding process occurring when ribosomes encounter a signal embedded in the mRNA of certain eukaryotic and prokaryotic genes. This signal has a mandatory component, the frameshift motif: it is either a Z_ZZN tetramer or a X_XXZ_ZZN heptamer (where ZZZ and XXX are three identical nucleotides) allowing cognate or near-cognate repairing to the -1 frame of the A site or A and P sites tRNAs. Depending on the signal, the frameshifting frequency can vary over a wide range, from less than 1% to more than 50%. The present study combines experimental and bioinformatics approaches to carry out (i) a systematic analysis of the frameshift propensity of all possible motifs (16 Z_ZZN tetramers and 64 X_XXZ_ZZN heptamers) in *Escherichia coli* and (ii) the identification of genes potentially using this mode of expression amongst 36 *Enterobacteriaceae* genomes. While motif efficiency varies widely, a major distinctive rule of bacterial -1 frameshifting is that the most efficient motifs are those allowing cognate re-pairing of the A site tRNA from ZZN to ZZZ. The outcome of the genomic search is a set of 69 gene clusters, 59 of which constitute new candidates for functional utilization of -1 frameshifting.

## INTRODUCTION

Programmed ribosomal -1 frameshifting (PRF-1) has been recognized more than 25 years ago as a mode of translational control of specific genes, first in retroviruses (1–3) and later in bacterial genes (4,5). Since then the number of demonstrated or suspected cases, has greatly increased, generally through homology searches, or by taking advantage of the many sequenced genomes to look for genes containing potential frameshift signals (6,7). For example, the Recode database (8) has 245 entries for -1 frameshifting originating from eukaryotic viruses (192 cases), from transposable elements (31 cases, 6 bacterial and 25 eukaryotic), from bacteriophages (12 cases) and from chromosomal genes (10 cases). Overall, 219 entries come from eukaryotic genes. This may give the impression that -1 frameshifting is less common in prokaryotes, but it is not necessarily true. Analysis of the ISFinder database (9), dedicated to bacterial transposable elements called insertion sequences (IS), showed that more than 500 IS elements very likely use -1 frameshifting to synthesize the proteins necessary for their mobility (10). Another bioinformatics study carried out on 973 bacterial genomes revealed more than 5000 genes that probably use -1 frameshifting (11). These genes can be grouped into a limited number of clusters most of which correspond to IS elements. Although, like their eukaryotic counterparts, most bacterial genes likely using programmed -1 frameshifting are found in mobile elements, such as IS transposons or bacteriophages (12), utilization of -1 frameshifting may not be limited to them. Another study revealed a set of 146 prokaryotic gene families with various potential programmed frameshifts, several of which were experimentally tested (13,14). Most of these families correspond to non-mobile genes encoding proteins of known functions and proteins with conserved domains performing yet unknown functions. Sequences of genes from these clusters are available from GenTack database (13).

**Figure 1.**
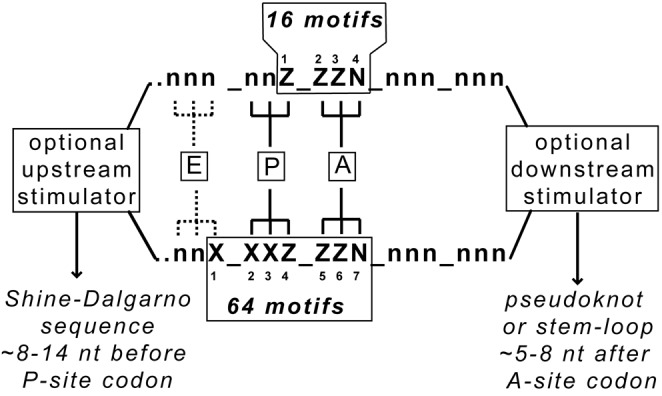
Overall organization of known bacterial -1 frameshift signals. Codons in frame with the upstream initiation codon (frame 0) are separated with underscores; their position relative to the ribosomal E, P and A sites and their tRNAs, at the onset of frameshifting is indicated.

**Figure 2.**
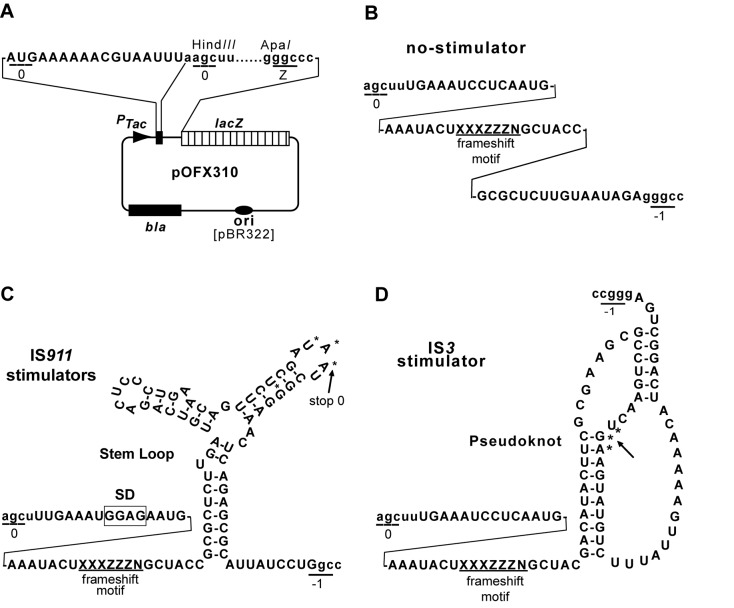
Reporter plasmid and sequence of the three contexts in which the frameshifting propensity of the X_XX.Z_ZZ.N heptamers was assessed. Plasmid pOFX310 (panel A) was used to clone between a Hind*III* and an Apa*I* site the three frameshift windows shown in panels B–D. The no-stimulator construct (panel B) was derived from the IS*911* construct (panel C) (48) by deletion of most of the stem-loop and mutation to CCUC of the SD-like GGAG sequence. The IS*3* construct (panel D) was engineered by replacing the IS*911* stem-loop with the PK from IS*3* (4) and by mutating to CCUC the stimulatory SD.

The execution of frameshifting at a significant level requires a relatively simple signal which is embedded within the coding part of certain mRNAs (Figure 1). The two components of this signal were revealed by the earlier studies on retroviruses (1–3). The obligatory component is a short sequence of 7 nucleotides, X_XX.Z_ZZ.N, called the frameshift motif or ‘slippery’ motif, where XXX and ZZZ, are triplets of identical bases, and N is any nucleotide (underscoring separates codons in frame with the initiation codon, i.e. frame 0, and dots separate codons in the new frame, i.e. frame -1). Thus, there are 64 possible sequences corresponding to the above definition. It was also shown that an even shorter sequence, a Z_ZZ.N tetramer (where ZZZ are three identical bases, thus leading to 16 possible motifs), could also direct programmed -1 frameshifting (15–21). The‘slipperiness’ of both types of motifs likely results from their capacity to allow cognate or near cognate re-pairing in the -1 frame of one or two tRNAs (2,22). On an X_XX.Z_ZZ.N heptamer, the XXZ- and ZZN-decoding tRNAs, respectively, in the P and A sites of the ribosome, would break the codon–anticodon interaction and re-pair on the XXX and ZZZ codons in the -1 frame. The second component of frameshift signals is a stimulatory element which, by itself, cannot induce frameshifting. It can be an RNA secondary structure, such as simple or branched hairpin-type stem-loop (HP) or a pseudoknot (PK) (23–25). As illustrated in panels C and D of Figure 2, it is formed by local folding of the mRNA and generally starts 5–8 nucleotides downstream of the slippery sequence (17,23,26). The stimulatory effect of a structure may be linked to its capacity to block ribosomes transiently when the ZZN codon occupies the A site and thus give more time to tRNAs for re-pairing (27–29). In addition, a structure may exert a pulling effect on the mRNA and favour its realignment within the ribosome to bring the XXX and ZZZ -1 frame codons in the P and A sites (30,31). It is present in all well-studied eukaryotes cases, often as a PK, but not always found in prokaryotes PRF-1 regions (10). Prokaryotic signals sometimes possess another type of stimulatory element upstream of the frameshift motif: a Shine–Dalgarno (SD)-like sequence normally involved in translation initiation through pairing with the CCUCC sequence at the 3′ end of 16S ribosomal RNA (32–34). In PRF-1 signals, the same interaction occurs but within an elongating ribosome and results in a translational pausing (35), which may provide a longer time window for tRNA re-pairing. The other possible effect of a stimulatory SD is linked to its distance from the motif which could generate a tension between the mRNA and the ribosome that could be resolved by realigning the mRNA (32). Thus, the two types of stimulators could act in concert by generating pausing, for both, and by pushing (SD) or pulling (structure) on the mRNA. In addition, frameshifting frequency in eukaryotes and prokaryotes is modulated by the immediate context on both sides of the slippery motif (36–38). It is not yet clear by which mechanism(s) this modulation operates but it could result in part from an E site tRNA effect (38), from intra-mRNA interactions (36) and possibly from mRNA–ribosome interactions within the message entry tunnel (39).

The slippery motif, being the key element in frameshifting, has been the object of a particular attention. An early study dealt with the motif present in the Rous sarcoma virus signal (2). Mutating it to a limited set of different motifs lead to the proposal of basic rules governing X_XX.Z_ZZ.N heptamers frameshifting efficiency for eukaryotes: in short, substantial frameshifting is attained if XXX = [AAA, GGG or UUU], ZZZ = [AAA or UUU] and N = [A, C or U]. A subsequent nearly systematic analysis confirmed these rules: 44 motifs were tested and the 20 remaining motifs were not included because of their expected inefficiency (see upper panel of Supplementary Figure S1A) (17). Thus, only a subset of the 64 possible X_XX.Z_ZZ.N heptamers can elicit frameshifting at a significant level in higher eukaryotes.

The X_XX.Z_ZZ.N heptamers were also analysed in prokaryotes, using the *Escherichia coli* bacterium, but not as thoroughly as in eukaryotes (19,36,40–42). It turned out that the most proficient motifs, the X_XX.A_AA.G heptamers, were the least efficient ones in eukaryotes. Conversely, the best eukaryotic motifs proved inefficient in *E. coli* (e.g. A_AA.A_AA.C or G_GG.A_AA.A), thus indicating major differences in the response of the respective translational machineries to these signals. However, the limited scope of these studies, in terms of number of motifs tested, did not allow the establishment of precise rules concerning slippery heptamers efficiency in bacteria. The first aim of the work presented here is to determine these rules by carrying out a complete functional analysis in *E. coli* of both types of potential frameshift motifs, the Z_ZZ.N tetramers and X_XX.Z_ZZ.N heptamers. The second objective is to investigate, by bioinformatics approaches, the prevalence of the X_XX.Z_ZZ.N motifs in 37 enterobacterial genomes, mostly from *E. coli* isolates, in order to determine whether or not they have been selected against in coding sequences because of their frameshifting proclivity. Our third aim is to identify genes possibly utilizing -1 programmed frameshifting by analysing, in the same set of genomes, those containing a subset of 21 heptamers, which were chosen on the basis of their -1 frameshifting efficiency and/or their significant underrepresentation.

## MATERIALS AND METHODS

### Bacterial strain, growth conditions and transposition assay

The *E. coli* K-12 strain JS238 [MC1061, *araD* Δ(*ara leu*) *galU galK hsdS rpsL* Δ(*lacIOPZYA*)*X74 malP*::*lacI*q *srlC*::Tn*10 recA1*] was used for all experiments. Bacterial cultures were carried out in Luria-Bertani (LB) medium (43) to which Ampicillin (40 mg/l) plus oxacillin (200 mg/l) were added when necessary.

### Plasmid constructions for assessing -1 frameshifting

All frameshift cassettes were cloned into the pOFX310 reporter (Figure 2A), derived from the pAN127 plasmid (40) by changing the translation initiation region of the *lacZ* gene, between the Xba*I* and Hind*III* to tctagCTCGAGATTTATTGGAATAACAT**ATG** AAA AAA CGT AAT TTa agc tt (the Xba*I* and Hind*III* sites are in lowercase, the SD sequence GGA and the ATG start codon in frame 0 are both underlined). Overlapping oligonucleotides were inserted between the Hind*III* and Apa*I* sites of the vector to reconstitute the various frameshift regions in front of the *lacZ* gene so that expression of β-galactosidase requires a -1 ribosomal frameshifting event within the cloned cassette. For each type of frameshift region (i.e. no stimulator, IS*911* stimulators or IS*3* stimulators, see Figure 2B–D), an in-frame construct was made to serve as 100% reference for calculation of frameshifting frequencies from β**-**galactosidase activities. A non-shifty derivative was constructed for each motif to assess the background level of frameshifting. The rationale was to keep the same tRNA in the A site (Z_ZZ.N motifs) or in both A and P sites (X_XX.Z_ZZ.N motifs). For the Z_ZZ.N tetramers only the first nucleotide was mutated to G_YY.N or C_RR.N (with Y = [U, C] and R = [A, G]). For the heptamers, the first and fourth nucleotides of the motifs were changed to give G_YY.C_UU.N, C_RR.C_UU.N, G_YY.U_CC.N, C_RR.U_CC.N, G_YY.G_AA.N, C_RR.G_AA.N, G_YY.A_GG.N or C_RR.A_GG.N.

### Measurement of frameshifting frequency by β-galactosidase assay

Transcription of lacZ relies on a strong, isopropyl-β-D-thiogalactopyranoside-inducible, pTac promoter. Its expression was monitored by a standard colorimetric assay (43) on cultures prepared either in the absence of inducer, for constructs with a sufficiently high level of β-galactosidase activity (i.e. above 0.2% frameshifting), or after isopropyl β-D-1 thiogalactopyranoside induction for the ones with a low activity (i.e. those with less than 11% frameshifting). For each strain, 5 tubes containing 0.5 ml of Luria-Bertani medium (supplemented with ampicillin and oxacillin) were inoculated with independent clones and incubated overnight at 37°C. These cultures were either diluted 1/250 in the same medium and incubated 270 min at 37°C (no-induction conditions) or diluted 1/50 in the same medium plus 2 mM of isopropyl-β-D-thiogalactopyranoside and incubated 240 min at 37°C (induction conditions). The dosage conditions were as previously described (19). Note that both methods gave identical % frameshifting values in their overlap range, i.e. between 0.2% and 11%. We also verified the accuracy of the reported values above or below the overlap range, by applying to a limited set of plasmid constructions a refined assay in which non-induced cultures were first concentrated and lysed by sonication (data not shown).

### Generation and randomization of a non-redundant ‘mostly *E. coli* genome’ (nrMEG)

The Refseq accessions of the genomes that were used to construct our nrMEG, together with their organism/strain information, are given in Supplementary Table S1. All the protein coding gene sequences were extracted from the .ffn files (National Center for Biotechnological Information website; ftp://ftp.ncbi.nlm.nih.gov/genomes/Bacteria/) of these 37 accessions and merged together to make a combined genome of 169 302 sequences. These sequences were clustered using the BLASTCLUST program using a 95% sequence identity threshold at the level of nucleotide sequence. One representative sequence per cluster was randomly chosen to constitute an nrMEG of 22 703 sequences. Each sequence from the nrMEG was randomized 1000 times using the Dicodonshuffle randomization procedure (44) to yield 1000 randomized nrMEGs. The DicodonShuffle algorithm preserves the dinucleotide composition, the encoded protein sequence and the codon usage of each gene.

### Analysis of XXXZZZN frequencies in protein coding sequences

A customized perl script was used to count the occurrences of a pattern in all three possible reading frames (i.e. X_XX.Z_ZZ.N, XX_X.ZZ_Z.N and XXX_.ZZZ._N) in both the real and randomized nrMEGs. Violin plots (45), generated with the vioplot package from the R software library (http://www.r-project.org), were used to visualize the occurrences of the 64 X_XX.Z_ZZ.N patterns. *Z*-scores were computed as follows: *z*-score = (*x* − *x*_mean_)/*x*_sd_, where *x* is the frequency of occurrence of a pattern in the integrated genome, *x*_mean_ is the mean of the distribution of the same pattern across 1000 randomized genomes and *x*_sd_ is the standard deviation of the distribution of the same pattern across 1000 randomized genomes. *Z*-scores for the 64 XXXZZZN in all three frames are shown in Supplementary Table S3 while all violin plots are available online at http://lapti.ucc.ie/heptameric_patterns_clusters/.

### Clustering of genes containing selected X_XX.Z_ZZ.N patterns

All the annotated protein coding genes from 36 of the 37 genomes listed in Supplementary Table S1 (AC_000091 was later excluded because of its removal from Refseq) were screened for the presence of a motif from a set of selected 21 X_XX.Z_ZZ.N patterns (see Results section). These sequences were clustered based on similarity between the encoded protein sequences using the BLASTCLUST program (sequence identity threshold = 45%). A total of 658 clusters which had at least 20 sequences and where the heptameric pattern was perfectly conserved were taken up for further analysis. The coordinates of the conserved heptameric patterns were also recorded for each cluster.

However, these clusters contain only sequences of protein coding genes from those 36 genomes, which were initially selected to constitute the nrMEG. These clusters were enriched with additional homologous sequences from the genomes not included in the nrMEG in an attempt to obtain a better phylogenetic signal. The enrichment was carried out using a tblastn search against all bacterial sequences in the nr database as described previously (11). The ‘newly’ obtained homologous sequences for each cluster were aligned by translating them into protein sequences, aligning these protein sequences and then back translating the aligned protein sequences to their corresponding nucleotide sequences. The coordinates of the conserved heptameric pattern for each cluster (which were recorded in the previous step) were recalculated to account for gaps introduced during alignment of additional sequences.

### Identification of clusters with conserved heptameric pattern

For the 658 clusters identified in the previous step, we employed an additional filtering procedure to identify those clusters where the heptameric pattern is conserved. For each cluster, the total number of sequences was referred to as N_all_. The number of sequences where the heptameric pattern was the same as the parent pattern was referred to as N_1_. The number of sequences where the pattern is not the same but is one of the 64 X_XX.Y_YY.Z patterns, was referred to as N_2_. Finally, it has been previously observed (e.g. in *dnaX*) that the position of the frameshift site may not be perfectly conserved. To account for that possibility, a 36-nt window starting from the coordinate which is 15 nt upstream of the conserved heptameric coordinate was also screened for the presence of any X_XX.Y_YY.Z pattern; the number of sequences in that category was referred to as N_3_. For each cluster, the three values were summed up (i.e. N_1_+N_2_+N_3_ = N_sum_) and the ratio N_sum_/N_all_ was calculated. Clusters where N_sum_/N_all_ > 0.9 were labelled as ‘conserved’. In an ideal situation, the frameshift pattern should be absolutely conserved, but this threshold was relaxed so as to allow for the possibility of sequencing errors or recent mutations in the sequences from a cluster. The end result was a set of 69 clusters (features of these clusters are presented in Supplementary Tables S4–S8, and the complete sequence of their genes and other features are available at http://lapti.ucc.ie/heptameric_patterns_clusters/).

### Synonymous-site conservation analyses

The degree of conservation at synonymous sites was calculated as previously described (46) for a 15-codon window. The detailed results of this analysis are available online at http://lapti.ucc.ie/heptameric_patterns_clusters/ and a summary is included in the RSSV column (for reduced variability at synonymous sites) of Supplementary Tables S4 and S5. However, a statistically significant conservation at synonymous sites can only be observed if there is sufficient sequence divergence in the alignment. To numerically quantify sequence divergence, we also calculated a statistic called aln_div_ which corresponds to an estimate of the mean number of phylogenetically independent nucleotide substitutions per alignment column (see Supplementary Tables S4 and S5).

### Search of potential stimulatory elements flanking the pattern

Two types of potential frameshift stimulators were searched. The first type was an SD-like sequence (either GAGG, GGAG, AGGA, GGNGG or AGGKG, with K = [T,G]) located 6–17 nt before the second base of the motif; these sequences and the spacing interval were chosen because all were experimentally proved to be stimulatory (32). A segment of 30 nt ending with the first base of the motif was scanned, using a script for Perl (version 5.10.1 from ActiveState), in all the sequences of each cluster for the above potential SD sequences. A cluster qualified as having a ‘conserved SD’ if at least 50% of its sequences had an SD.

The second type of stimulator was an RNA structure downstream of the motif. A preliminary study of 271 IS*3* family members (see Supplementary Figure S2) led us to choose the following empirical rules: the structure is (i) a simple or branched hairpin of a length ranging from 17 to 140 nt, (ii) that starts 4–10 nt after the last base of the motif, (iii) with a G-C(or C-G) base-pair followed by at least three consecutive Watson–Crick or G–U or U–G base-pairs and (iv) has a ΔG_unfold@37°C_ ≥ 7.6 kcal.mol^−1^; the ΔG_@37°C_ value was determined using the default parameters of the RNAfold program from version 1.8.5 of the Vienna RNA package (47). We limited our search to hairpin structures and and did not explore whether some of the structures could also form PKs at this preliminary stage. For each sequence of all clusters, a 197 nt segment starting at the fourth base after the motif was extracted and analysed with a custom Perl script. Each segment was first deleted from the 3′ end one base at a time and down to 17 nt. Each set of nested deletions was passed to the RNAfold program and the potential structures were sorted out to retain those conforming to the rules. For a given cluster, structures were grouped in types according to hairpin size and distance from the motif. The frequency of each type of structure was calculated and only those present in at least 50% of the sequences of the cluster were retained; 53 of the 69 clusters had such a conserved structure. The ΔG_hp_.nt^−1^ parameter (i.e. the ΔG_unfold_ value divided by the number of nucleotides in the hairpin structure) was calculated. For clusters with several types of structure, the ‘best’ type was the one with the highest value for the [(ΔG_hp_.nt^−1^) x (frequency)] product. A summary of these analyses is reported in Supplementary Tables S4, S5 and S8.

For further comparison, 20 ISs from the IS3 family were selected because they contain a frameshift motif followed by a known (or likely) stimulatory structure of a size ranging from 17 to 131 nt. The ΔG_hp_ and ΔG_hp_.nt^−1^ parameters were calculated for each structure. Selective pressure to maintain a hairpin should likely result in a region more structured than neighbouring regions of the same size, i.e. having a value higher than average for the ΔG.nt^−1^ parameter. To assess that, a 197 nt segment starting 4 nt downstream of the frameshift motif was extracted for each of the 20 ISs as well as for one typical sequence of each of the 53 clusters with a conserved structure. For each 197 nt segment, a Perl script generated a subset of sequences by moving (1 nt at a time) a sliding window of the size of the corresponding conserved hairpin and passed it to RNAfold. The average ΔG_unfold_.nt-1 (ΔG_av_.nt^−1^) were calculated for each subset as well as the ΔΔG.nt^−1^ which is the difference [(ΔG_hp_.nt^−1^) − (ΔG_av_.nt^−1^)]. As expected, the 20 ISs possessing a stimulatory structure all display a higher than average ΔΔG.nt^−1^ (Supplementary Table S8).

## RESULTS

### *In vivo* determination of -1 frameshifting frequency

As illustrated in Figure 2, the 16 Z_ZZ.N motifs and the 64 X_XX.Z_ZZ.N motifs were cloned either without flanking stimulators, or with a strong downstream stimulator derived from the IS*3* PK (18,19), or, for the heptamers only, with the moderately efficient combination of upstream and downstream stimulators from IS*911* (32,48). For both types of motifs, a non-shifty derivative was similarly cloned: for that, the first base of each tetramer or the first and fourth bases of each heptamer were mutated. The shifty and non-shifty cassettes were inserted in front of the *lacZ* gene, carried by plasmid pOFX310, so that translation of full-length β-galactosidase occurs only when ribosomes move to the -1 frame before encountering the 0 frame stop codon (Figure 2).

### Frameshift propensity of the Z_ZZ.N motifs

**Figure 3.**
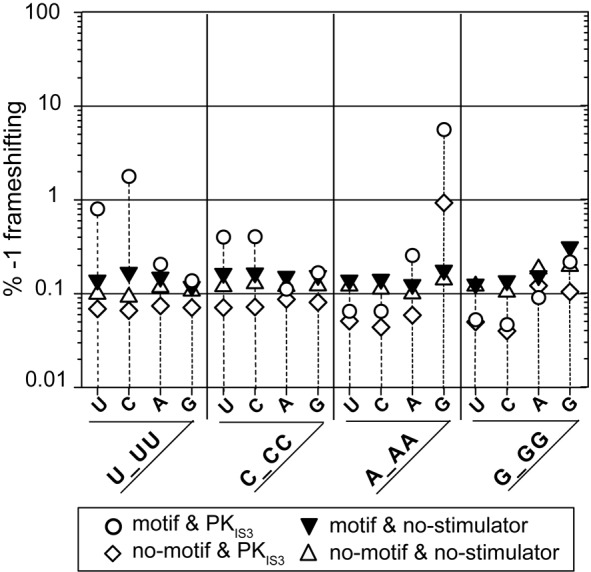
Frameshift efficiency of the Z_ZZ.N tetramers. The IS*3* frameshift region cloned in plasmid pOFX310 was the one used in a previous study [see Figure 1 in (19)]. It differs slightly from the one used for the heptamer analysis (Figure 2, panel D). The nucleotides upstream (6 nt) and downstream (5 nt) of the motif are those found in IS*3*. The sequence from the Hind*III* site to the start of the PK is agcuuCCUCCA**ZZZN**GCCGC—. The no-stimulator construct was derived by deleting the 3′ half of the PK, right after the UGA stop codon in the 0 frame, to give the following sequence: agcuuCCUCCA**ZZZN**GCCGCGACAUACUUCGCGAAGGCCUGAACUUGAAgggcc. The four frameshifting values for each motif correspond to a construct with a motif and the IS*3* PK (open circles), a construct without motif and with the IS*3* PK (open lozenges), a construct with motif and without stimulator (black inverted triangles) and a construct without motif and stimulator (open triangles). Each frameshifting value is the mean of five independent determinations (the ± standard deviation intervals were omitted because they are not bigger than the size of the symbols in most cases). The no-motif constructs were derived by changing each motif to either G_YY.N or C_RR.N.

The graphs showing the variation of -1 frameshifting frequency as a function of the sequence of the motif are presented in Figure 3 for the Z_ZZ.N tetramers (see also Supplementary Table S2). The motif-containing constructs without PK (save G_GG.G, see below) were on the average marginally above their no-motif counterpart (0.147 ± 0.004% versus 0.112 ± 0.015%), which suggests that the motifs are by themselves barely or not at all shifty. Addition of the IS*3* PK led to substantial increase in frameshifting frequency for 10 motifs. Only six were at least four times above background (i.e. above 0.064 ± 0.014%), with frequencies ranging from 0.26% to 5.6%. For them the hierarchy was [A_AA.G >> U_UU.C > U_UU.U > C_CC.U = C_CC.C> A_AA.A]. Four motifs (U_UUA, U_UUG, C_CCA and C_CCG) were 1.8- to 3.6-fold above background.

A few oddities were revealed. The G_GG.G, C_GG.A and C_GG.G constructs (with and without PK) were found above background probably not as a result of frameshifting but because these sequences, together with the following G, could act as SD sequences and direct low level initiation on the -1 frame AUA codon present 7 nt downstream (see legend of Figure 3). The other oddity, C_AA.G, was nearly 10 times above background (0.93%) but only when the PK stimulator was present: this was likely due to -1 frameshifting caused by the high shiftiness of the lysyl-tRNA_UUU_ (41,49) combined to the high efficiency of the PK stimulator.

### Frameshift propensity of the X_XX.Z_ZZ.N motifs

**Figure 4.**
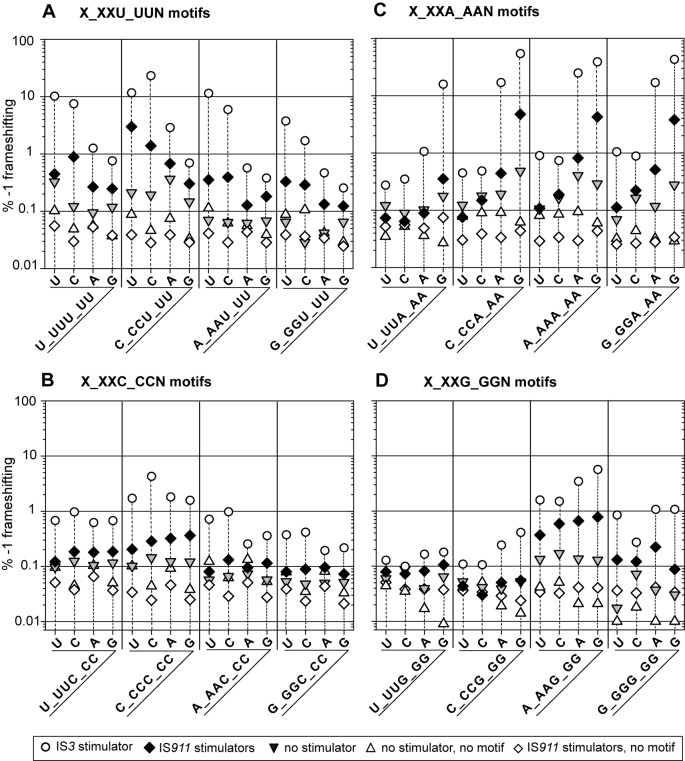
Frameshift efficiency of the X_XXZ_ZZN heptamers. There are five frameshifting values for each motif corresponding to constructs with a motif and the IS*3* PK (open circles), with a motif and the IS*911* stimulators (black lozenges), without a motif and with the IS*911* stimulators (open lozenges), with a motif and without stimulator (inverted grey triangles) and without both motif and stimulator (open triangles) (Figure 2). Each frameshifting value is the mean of five independent determinations (the ±standard deviation intervals were not added because they are not bigger than the size of the symbols). The no-motif constructs were obtained by changing the first and fourth nucleotides of each motif as detailed in Materials and Methods.

The results obtained for the X_XX.Z_ZZ.N heptamers and their non-shifty derivatives are presented in Figure 4. The average background values given by the no-motif constructs was 0.037 ± 0.010% for those with the IS*911* stimulators (open lozenges) and 0.055 ± 0.031% for those without stimulator (open triangles). The estimated background value for the IS*3* constructs was of 0.069 ± 0.007% (data not shown). The frameshifting frequency among the motif-containing constructs varied over a large range, i.e. from 0.018% for G_GG.G_GG.U without stimulator to 54% for C_CC.A_AA.G associated with the IS*3* PK. As previously demonstrated in *E. coli*, the most efficient motifs, in the presence of the IS*911* or IS*3* stimulators, were C_CC.A_AA.G, G_GG.A_AA.G and A_AA.A_AA.G (19,40,41). The ratio between the motif and no-motif frameshifting frequency values was used as a classifier of motif efficiency: motifs displaying a ratio above 2 were categorized as frameshift-prone. Among the constructs without stimulator, 39 motifs (61%) met this criterion (ratio from 2.1 to 10). When the IS*911* stimulators were added, 55 motifs (86%) showed a ratio ranging from 2.1 to 112. Swapping the moderate IS*911* stimulators for the more efficient IS*3* PK, increased further the motif to no-motif ratio (from 2.1 to 1188) and raised the number of positive motifs to 61 (95.3%); the 3 motifs below the threshold were A_AA.C_CC.A, G_GG.C_CC.A and C_CC.G_GG.C.

In spite of divergences as to its timing in the elongation cycle, the general view concerning -1 frameshifting on slippery heptamers is that it occurs after proper decoding of the ZZN 0-frame codon when the P and A ribosomal sites are occupied by the XXZ and ZZN codons and their cognate tRNAs (2,22,30,40,50,51). Simple rules emerge when the effect of the ZZN codon is considered (Figure 4). The UUN and AAN codons are on the average more frameshift-prone than CCN and GGN. Whatever Z is, the two homogeneous ZZN codons (meaning all purine or all pyrimidine bases) are better shifters than the two corresponding heterogeneous ones. To explain further all the variations in frameshifting frequency, it is also necessary to take into account the nature of the X nucleotide. Motifs are by and large more frameshift-prone when the X and Z nucleotides are homogeneous, i.e. all purines or all pyrimidines. Notable exceptions to the latter rule are Y_YY.A_AA.R and R_RR.U_UU.Y (with Y = [U, C], R = [A, G]). Here, the high shiftiness of the AAR and UUY codons probably counteracts the negative effect of the YYA and RRU heterogenous codons.

### Distribution of frameshift motifs in IS elements

**Figure 5.**
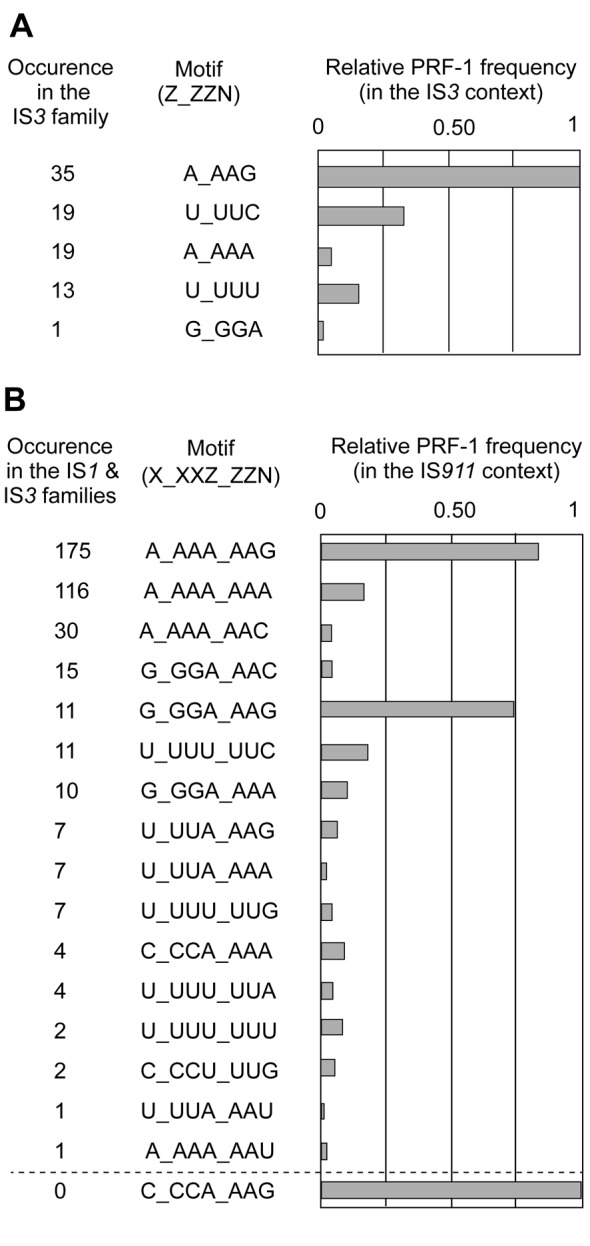
Distribution of Z_ZZ.N and X_XX.Z_ZZ.N motifs in mobile elements from the IS*1* and IS*3* families. These two families were selected because biologically relevant -1 frameshifting was demonstrated in both (4,10). The sequences of the IS from these 2 families (63 entries for the IS*1* family and 494 for the IS*3* family), obtained from the ISFinder database (October 2012), were examined for the presence of potential frameshift signals (i.e. existence of 2 overlapping ORFs, with the second being in the -1 frame relative to the first and presence of a Z_ZZ.N or X_XX.Z_ZZ.N motif in the overlap region; the Z_ZZN motifs scored in panel B are those which are not part of an X_XX.Z_ZZ.N heptamer). The relative frameshifting frequencies of the motifs found are indicated in the right-hand panels. All values were normalized relative to that of the best motif (A_AA.G or C_CC.A_AA.G), using data from Figures 3 and 4.

From the above experimental study, we concluded that a majority of heptamers (and nearly half of the tetramers) were capable of eliciting -1 frameshifting at substantial levels (at least twice the background level). To determine the range of motifs used in genes utilizing frameshifting for their expression, we carried out an analysis of IS mobile genetics elements known, or suspected, to use this mode of translational control. We focused on the members of the IS*1* and IS*3* families available in the ISFinder database in October 2012 (9). As shown in Figure 5, both tetramers and heptamers are found, but with a marked preference for heptamers (87 against 403). Among the five tetramers, the three most shift-prone motifs, A_AA.G and U_UU.[U,C], predominate and the less efficient A_AA.A motif is also well represented. Only 16 different heptamers are found with 72% of them being either A_AA.A_AA.G or A_AA.A_AA.A. The next most frequent are A_AA.A_AA.C and G_GG.A_AA.C, both of low efficiency, followed by the more efficient G_GG.A_AA.G, U_UU.U_UU.C and G_GG.A_AA.A. To conclude, it appears that genes known or suspected to use PRF-1 to express a biologically important protein do not necessarily utilize high efficiency motifs. However, this conclusion is based on one category of genes where two overlapping genes code for the proteins required for transposition of two types of IS elements. There, the purpose of frameshifting is to provide the ‘right’ amount of a fusion protein which has the transposase function (18,52); this amount is what keeps transposition of the IS at a level without negative effect on the bacterial host. If the ‘right’ amount is a low amount, then the use of low-efficiency motifs, with or without flanking stimulators, is a way to achieve this goal as illustrated by the IS*1* element (53).

### Distribution of frameshift motifs in *E. coli* genes

Our objective was to statistically assess the prevalence of each X_XXZ_ZZN motif in the genome of various *E. coli* strains. The rationale was that if a given motif induces by itself frameshifting at a significant level (i.e. at a biologically detrimental level), then it should be counterselected and, therefore, be underrepresented in *E. coli* genes.

- **Generation of a non-redundant nrMEG**. In a recent study, 61 bacterial genomes, including strains of *E. coli*, *Shigella* and *Salmonella*, were compared to identify gene families that are conserved across all the genomes (core-genome; 993 families) and gene families which are specific to a particular genome (pan-genome; 15 741 families) (54). Our initial data set is somewhat inspired by this study. Sequences of all protein coding genes from 37 genomes (28 *E. coli*, 1 *E. fergusonii*, 7 *Shigella* and 1 *Salmonella*; Refseq accessions were available for only 37 genomes from the 61 mentioned in the study; see Supplementary Table S1) were first combined in a single file set, comprising 169 302 sequences, to form what we call a MEG. Sequences which shared more than 95% identity were clustered together to remove redundancy and only one representative sequence (randomly chosen) from each of these clusters was retained. This resulted in a significantly smaller nrMEG of 22 703 sequences.
- **Frequency of occurrences of heptameric patterns in the nrMEG.** The frequency of occurrences of each of the 64 X_XXZ_ZZN patterns in the nrMEG was determined. Additionally, the frequency of occurrences of the same pattern in the two other frames (XX_XXZ_ZN and XXX_ZZZ_N) was also determined. As a result, a total of 192 frequency counts (64 × 3) were obtained for the nrMEG.
- **Randomization of the nrMEG.** To determine whether adverse selection acting on a heptamer is due to their shifty properties and not due to other selective pressures such as mutational bias, codon usage or compositional bias of protein sequences, it is necessary to estimate how other selective pressures affect heptamer frequencies. For this purpose we used the Dicodonshuffle randomization procedure (44). Each gene sequence in the nrMEG was randomized 1000 times. This gave rise to a set of 1000 randomized enterobacterial genomes where each constituent gene sequence encodes for the same protein sequence, and has the same codon usage and dinucleotide biases as in the native nrMEG. Hence, the frequency of a heptamer's occurrence in these genomes could be used as an estimate of its frequency in the absence of selective pressure due to shift-prone properties of this pattern.
- **Comparison of the observed and expected values of the frequency counts for each pattern**. The frequency of occurrences of each of the 64 XXXZZZN patterns in each of the three possible frames (i.e. X_XXZ_ZZN, XX_XXZ_ZN and XXX_ZZZ_N) was determined across the 1000 randomized nrMEGs. Each pattern is represented by two numerical values: the mean and the standard deviation of its frequency distribution count across the 1000 randomized nrMEGs. To quantify the degree of under- or overrepresentation of each pattern we used a *z*-score (see Material and Methods section). A negative *z*-score implies that a pattern is underrepresented while a positive *z*-score is indicative of its overrepresentation (Supplementary Table S3). Under our assumption, we would expect only the ‘in-frame’ shifty motif (X_XXY_YYZ) and not the ‘out-of-frame’ shifty motifs (XX_XYY_YZ and XXX_YYY_Z) to be underrepresented.

**Figure 6.**
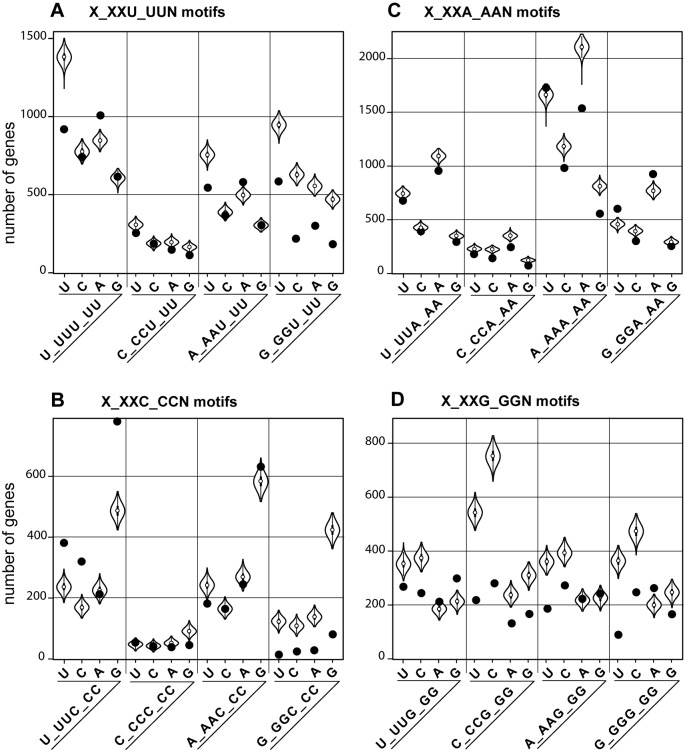
Distribution of the X_XXY_YYZ heptameric patterns across the nrMEG (black discs) and their spread across the 1000 randomized genomes (violins). The violin plots are a combination of a box plot and a kernel density plot where the width of the box is proportional to the number of data points in that box (45). The open circle in each violin correspond to the median, the thick vertical lines (often masked by the open circle) around the median represent the Inter Quartile Range while the thinner vertical lines that run through most of the violin plot represent 95% confidence intervals.

The comparison of each X_XXZ_ZZN pattern frequency in the nrMEG with its distribution in the randomized nrMEGs is shown in Figure 6. Black dots correspond to the number of occurrences of a particular motif in the nrMEG while the associated violin shows the distribution of the same motif across the randomized nrMEGs. Comparison with the *in vivo* data of Figure 4 shows that some of the sequence patterns, which are characterized by a marked underrepresentation, are also associated with high frameshifting efficiency (e.g. A_AA.A_AA.G). Two motifs, AAAAAAA and UUUUUUU, are notably underrepresented in all three frames (Supplementary Table S3). Possible reasons are that these patterns may interfere with gene expression in a frame-independent manner, producing indels in mRNA due to transcriptional slippage (55) or indel mutations at a high rate (56). Two motifs, the poor frameshifters C_CC.G_GG.C and C_CC.G_GG.U, are notably underrepresented and one motif, U_UU.C_CC.G, is markedly overrepresented. Frameshifting is not the only factor that may affect evolution of codon co-occurrence. It is possible that a particular pair of codons is slow to decode or results in ribosome drop-off. Such factors result in codon pair bias. The CCC_GGY and UUC_CCG codon pairs were indeed shown to be less frequent than expected for the formers and more frequent for the latter (57). The violin plots showing the comparison of patterns occurrence in the nrMEG and their distribution in the randomized nrMEGs in all three frames are available online at http://lapti.ucc.ie/heptameric_patterns_clusters/.

**Figure 7.**
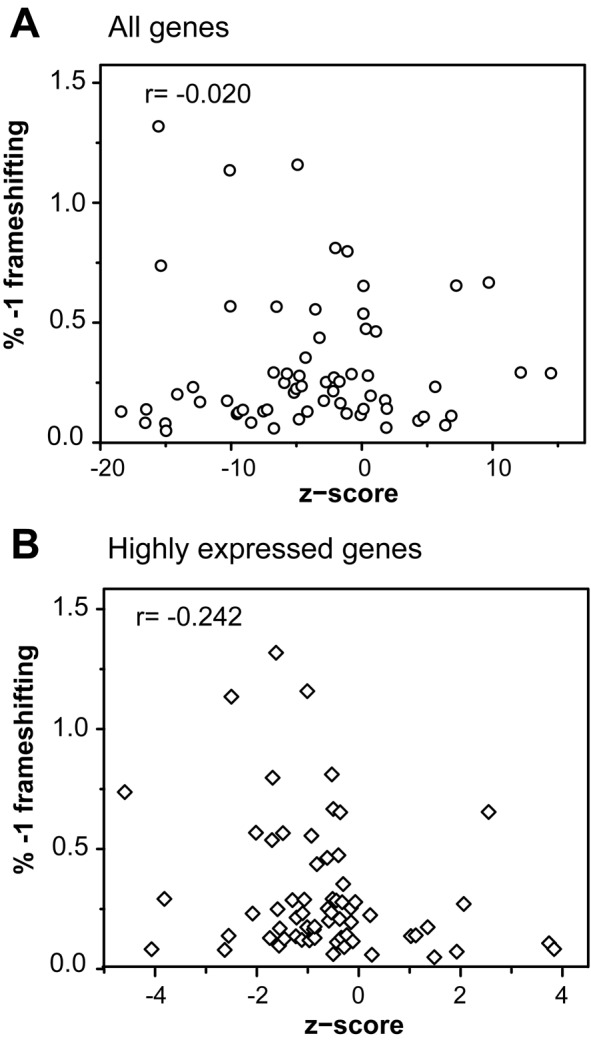
Plot of the z-score for all X_XX.Z_ZZ.N motifs in the nrMEG (panel A) or in the HEGome (panel B) against the frameshifting efficiency in the absence of stimulatory element. Note that the much larger *z*-values observed in (A) compared with (B) result from the much larger gene set being analysed in the former (leading to lower relative errors in what is essentially a Poisson system).

We anticipated that X_XX.Z_ZZ.N patterns characterized as shift-prone in our assays would be underrepresented due to selection pressure. Therefore, we expected to find negative correlation between *z*-scores and observed frameshifting efficiencies (in the absence of a stimulator) for these patterns. Surprisingly, no significant anticorrelation was found between the two measures (*r* = −0.020, *P* = 0.874; Figure 7A). Previously, underrepresentation of one shift prone pattern (A_AA.A_AA.G) was found to be more pronounced in highly expressed genes than in lowly expressed genes (6). Therefore, the distribution of X_XX.Z_ZZ.N motifs was analysed among 253 genes predicted as highly expressed in *E. coli* K12 (HEG database; http://genomes.urv.cat/HEG-DB/). These sequences were similarly randomized (10 000 times instead of 1000 because of the smaller size of this data set in comparison to the nrMEG set) and *z*-scores for each of the 64 patterns were computed. However, the correlation coefficient still remained non-significant albeit only marginally (*r* = −0.242, *P* = 0.054; Figure 7B).

### Search of genes possibly utilizing -1 programmed ribosomal frameshifting

The objective was to identify genes likely using PRF-1 on the basis of several criteria: (i) presence of an efficient motif (defined below), (ii) conservation of this motif (or a very similar one) in a given family of homologous genes from 36 selected genomes (see Supplementary Table S1 and Materials and Methods) and even beyond, in orthologous sequences, (iii) sequence conservation around the motif, (iv) presence of potential stimulatory elements flanking the motif and (v) position of the motif in the gene and consequence of frameshifting in terms of protein products [i.e. synthesis of a shorter or of a longer hybrid protein; note that the answer does not provide evidence for or against frameshifting since there are proven PRF -1 cases leading to one or the other outcome (12)].

We selected a subset of 18 X_XX.Z_ZZ.N patterns with a negative *z*-score and an *in vivo* frameshifting efficiency of more than 0.10% in the absence of stimulators listed below.

**Table:** 

A_AA.A_AA.A	A_AA.A_AA.G	A_AA.A_AA.C	A_AA.G_GG.C
A_AA.G_GG.U	C_CC.A_AA.A	C_CC.A_AA.G	C_CC.C_CC.A
C_CC.C_CC.G	C_CC.U_UU.A	C_CC.U_UU.G	C_CC.U_UU.U
G_GG.A_AA.G	U_UU.A_AA.G	U_UU.A_AA.U	U_UU.C_CC.A
U_UU.U_UU.C,	U_UU.U_UU.U.		

Three non-underrepresented patterns (C_CC.U_UU.C, A_AA.G_GG.A and A_AA.G_GG.G) were also considered because they exhibit high level frameshifting.

**Figure 8.**
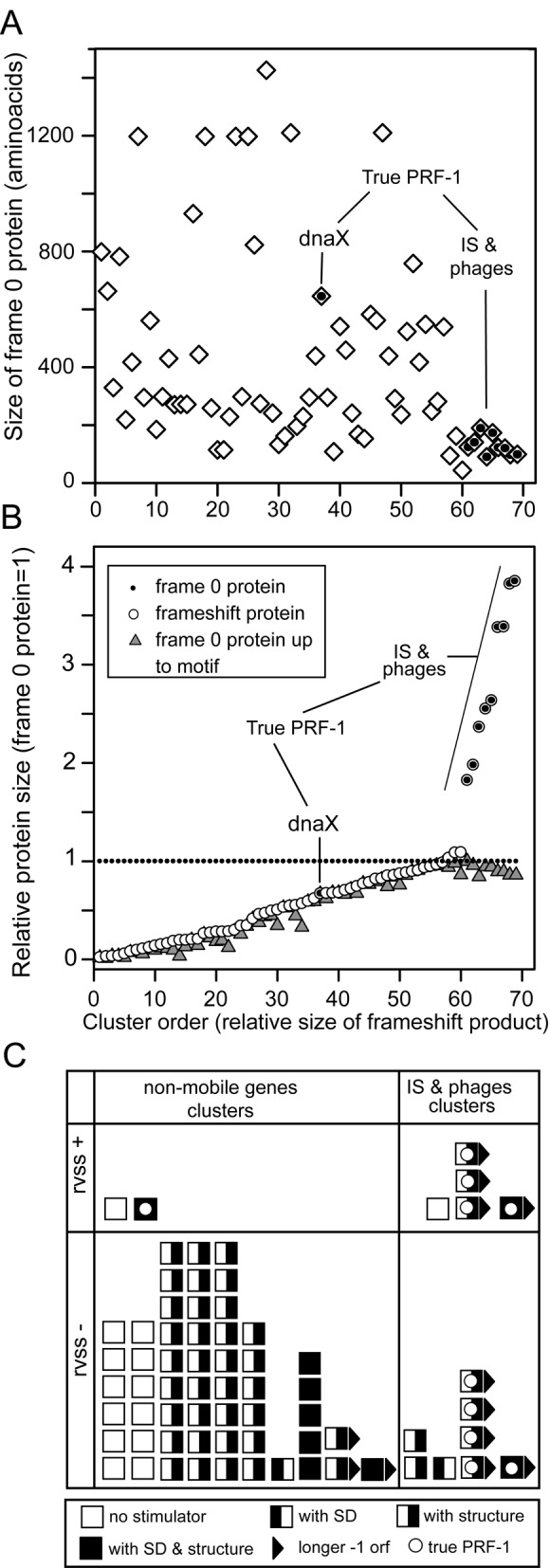
Overview of the proteins produced by normal translation or by -1 frameshifting for one gene typical of each of the 69 clusters of genes selected on the basis of high conservation of an X_XX.Z_ZZ.N motif in the nrMEG. The selection pipeline is indicated in Materials and Methods and the properties of each cluster are given in Supplementary Table S4–S6. The data of Supplementary Table S6 relative to the size of the protein products were plotted as follows. Panel A shows the size in amino acids of the full-length frame 0 protein as a function of the cluster order presented in the first column of Supplementary Table S6 (clusters were ordered by increasing value of the size ratio between the frameshift product and the frame 0 protein). Panel B, presents the size variation of the -1 frameshift product (circles) and of the normal, frame 0, translation product up to the end of the X_XX.Z_ZZ.N motif (triangles) (all sizes are relative to that of the corresponding full-length frame 0 product) as a function of the cluster order shown in the first column of Supplementary Table S6. The 10 demonstrated cases of frameshifting are indicated on each panel as true PRF-1 and *dnaX* or IS and phages (details about these clusters can be found in Supplementary Tables S4 and S5). Panel C summarizes qualitatively the features of each of the 69 clusters. Each square represents a cluster, and the features (absence of stimulator, presence of an upstream SD or of a downstream structure, existence of a -1 protein longer than the 0 frame product and demonstration of -1 PRF) are symbolized as indicated below the panel. Clusters in the two upper boxes are those displaying reduced variability at synonymous sites (rvss^+^; see Materials and Methods, Supplementary Tables S4 and S5) and clusters in the two lower boxes are those without reduced variability (rvss^−^).

Gene families possibly using these patterns for -1 PRF were identified using the pipeline described in Materials and Methods. This procedure led to 658 alignments which represented gene families with sequences containing one of the 21 chosen X_XX.Z_ZZ.N patterns. Subsequent filtering on the basis frameshift site conservation reduced that number to 69 clusters: 8 correspond to mobile genetic elements, 5 are from prophage genes and 56 belong to other gene families (Supplementary Table S4 and S5). The main features of these 69 clusters are summarized in Figure 8. It appears that the size of the gene containing the frameshift signal is very variable, since it can code for a 44–1426 amino acid protein (Supplementary Table S6, Figure 8A). In 57 clusters, the frameshift product is shorter than the product of normal translation (Supplementary Table S6, Figure 8B). The degree of conservation of synonymous sites around the frameshift site was also analysed (46); Figure 8C (http://lapti.ucc.ie/heptameric_patterns_clusters/). Synonymous sites are supposed to evolve neutrally unless there are additional constraints acting at the nucleotide sequence level, for example, pressure to conserve an RNA structure. Only 2 out of the 56 non-mobile genes display reduced variability at synonymous sites in the vicinity of the frameshift site, whereas 4 IS clusters and 1 prophage cluster do show such suppression (RVSS/aln_div_ column in Supplementary Tables S4 and S5). However, failure to detect statistically significant synonymous site conservation in the other clusters may be due to insufficient sequence divergence (RVSS/aln_div_ column in Supplementary Tables S4 and S5). Among the clusters displaying reduced variability, 1 non-mobile cluster (A_AAA_AAG_6), and 3 IS clusters (A_AAA_AAG_2, A_AAA_AAG_3 and A_AAA_AAG_4) possess a proven or potential stimulatory structure downstream of the motif. One IS cluster with reduced variability (A_AAA_AAC_1, a proven case of frameshifting) has no established stimulator (4,53). Two IS clusters do not display reduced synonymous site variability (A_AAA_AAA_1 and A_AAA_AAG_37) in spite of being proven cases where -1 frameshifting is stimulated by a stem-loop structure (unpublished data) (58).

**Figure 9.**
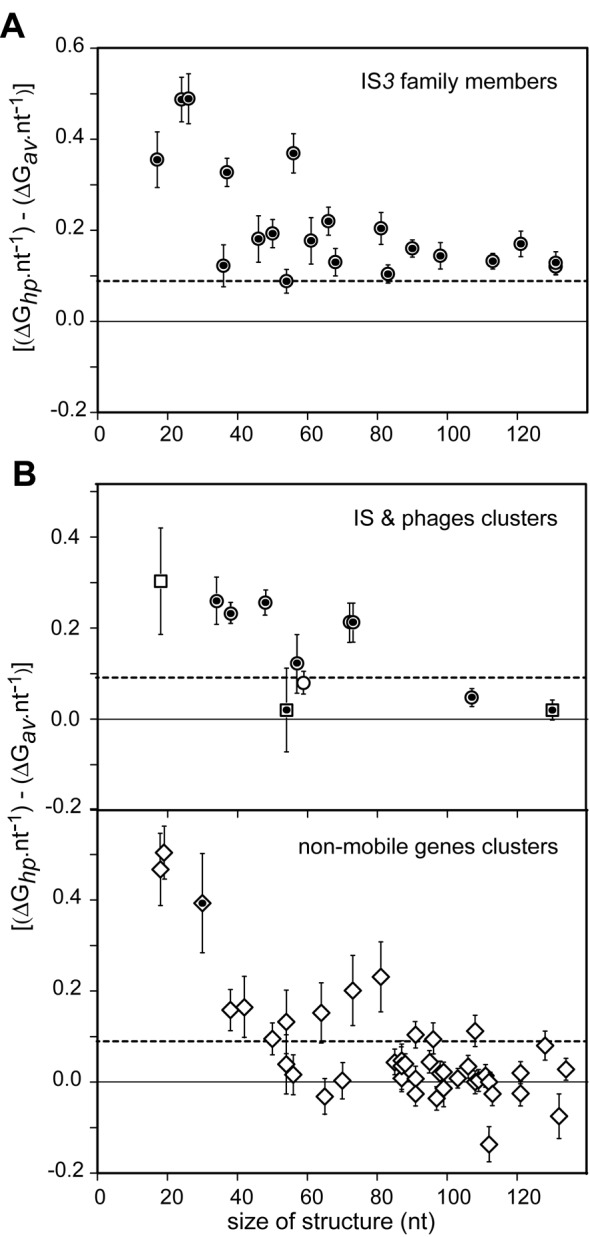
Summary of the *in silico* search of conserved hairpin structures constituting potential frameshift stimulators (see Materials and Methods). The *x-*axis indicates the size in nucleotide of the hairpin structure and the *y-*axis shows the ΔΔG.nt^−1^ parameter, which is the difference between the mean ΔG_unfold_ per nucleotide of the conserved hairpin (ΔG_hp_.nt^−1^, kcal.mol^−1^.nt^−1^) and the average ΔG_unfold_ of structures predicted in a sliding window, of the same size as the corresponding conserved hairpin, moved over a 197 nt segment starting 4 nt after the motif (ΔG_av_, kcal.mol^−1^) (see also Supplementary Table S8); the error bar correspond to the standard deviation for that difference. The symbols with a central black dot indicate genes for which PRF-1 is demonstrated or very likely. Part A shows the results for a set of IS*3* family members, namely (and ranked according to the size of the structure): IS*3411*, IS*Aca1*, IS*Susp2*, IS*3*, IS*1221A*, IS*Bcen23*, IS*Pae1*, IS*Psy11*, IS*Bmu11*, IS*Posp5*, IS*Bcen22*, IS*Bam1*, IS*Hor1*, IS*Xca1*, IS*L1*, IS*Dde4*, IS*Blma5*, IS*Spwi1*, IS*Nisp3* and IS*Rle5*. The dotted line is set at the lowest ΔΔG.nt^−1^ value (0.09 kcal.mol^−1^.nt^−1^) found for IS*Psy1*. Part B shows the ΔΔG.nt^−1^ results for 53 gene clusters, out of the 69, that have a conserved hairpin. The IS (circles) and phage (squares) clusters are in the upper panel and the non-mobiles genes clusters in the bottom panel.

In addition, the region 30 nt upstream of the motif was checked for the presence of a conserved SD-like sequence and the region extending 200 nt downstream of the frameshift site was analysed for the presence of a conserved RNA secondary structure; our criteria for a conserved stimulator was the presence of such a structure in at least 50% of the genes of a cluster. The SD-like sequences to be searched 6–17 nt upstream of the motif were those for which a stimulatory effect was experimentally demonstrated (Materials and Methods) (32). A conserved SD was found in 8 out of the 56 non-mobile clusters and in 3 out of the 13 IS and prophage clusters (Figure 8C, Supplementary Tables S4 and S8). In contrast, a potential stimulatory structure was predicted in a larger proportion of clusters: a conserved hairpin is present in the 8 IS clusters, in 3 out of 5 of the phage clusters and in 42 out of the 56 non-mobile genes clusters (see Materials and Methods for the parameters used to define the hairpin structure). Nine clusters possess both types of stimulators. To characterize further the predicted structures, we compared them with IS*3* family members possessing a frameshift site and an associated stimulatory structure (9,10). To assess structures of different sizes, we used a single parameter, ΔG_hp_.nt^−1^; which is the ΔG_unfold@37°C_ value of the hairpin divided by the number of nucleotides in the structure. An overall comparison showed that taken together the hairpins of our 53 clusters had a lower ΔG_hp_.nt^−1^ than those from a set of 271 IS*3* family members (0.317 ± 0.114 versus 0.452 ± 0.124 kcal.mol^−1^.nt^−1^; Supplementary Table S8 and Figure S2). For a more refined comparison, 20 IS*3* members were selected because they have a hairpin ranging from 17 to 131 nt. The average ΔG.nt^−1^ (ΔG_av_.nt^−1^) downstream of the frameshift motif was determined as detailed in Materials and Methods for these ISs as well as for our 53 clusters. The difference between ΔG_hp_.nt^−1^ and ΔG_av_.nt^−1^, ΔΔG.nt^−1^, was calculated and plotted against the size of the structure (Figure 9). It appeared that all the IS hairpins have a positive ΔΔG.nt^−1^ value (≥0.09 kcal.mol^−1^.nt^−1^) indicating that the hairpin segment is more structured than average, as expected if there is selective pressure for its maintenance (Figure 9A). The distribution of ΔΔG.nt^−1^ values is clearly not the same for our 53 clusters, especially the non-mobile genes clusters (Figure 9B): only 14 of them are at or above the 0.09 kcal.mol^−1^.nt^−1^ threshold value defined by the IS set. The remaining 28 clusters, as well as 2 phage clusters and 1 IS cluster, appeared to have a local folding level close or even below average. This suggests that their respective potential hairpins may not have been selected for but are fortuitous, non-biologically relevant, structures.

## DISCUSSION

### Comparison and meaning of prokaryotic and eukaryotic frameshifting rules

We determined that in *E. coli*, the rules of frameshifting on Z_ZZ.N tetramers are, in terms of motif hierarchy, A_AA.G > U_UU.Y > C_CC.Y >A_AA.A, the 10 remaining motifs were found barely or not at all frameshift-prone in our conditions. Thus, maintenance of a cognate tRNA-codon interaction after re-pairing of the A-site tRNA in the -1 frame is important to ensure efficient frameshifting. Notably, the maximal level of frameshifting remained about 10-fold lower than observed with the best heptamer associated with the same stimulatory element. In contrast, also with the heptamers, frameshifting on the Z_ZZ.N motifs definitely requires presence of a strong stimulator (Figure 3).

Relative frameshifting frequencies of 44 X_XX.Z_ZZ.N heptamers, tested in a eukaryotic system (rabbit reticulocytes lysate) (17) or in *E. coli* (Figure 4), are displayed in Supplementary Figure S1A; the motifs were placed upstream of stimulatory elements of similar efficiency, the avian infectious bronchitis virus (IBV) PK for the eukaryotic assay and the IS*3* PK for the *E. coli* series. A third of the motifs were not experimentally tested in the eukaryotic context because they were expected to be inefficient. Nevertheless, major rules of -1 frameshifting efficiency, as a function of the identity of the X, Z and N nucleotides, can be formulated (Supplementary Figure S1B). It appears that the most efficient motifs are D_DD.A_AA.H, D_DD.U_UU.H, U_UU.U_UU.G and C_CC.U_UU.U in the eukaryotic system and V_VV.A_AA.R, H_HH.U_UU.Y, U_UU.A_AA.G, C_CCC_CCC and A_AA.G_GG.G in *E. coli* (with D = [U, A, G], H = [U, C, A], R = [A,R], Y = [U,C] and V = [C, A, G]). The outcome is a slightly larger number of high efficiency motifs in the eukaryotic situation than in the bacterial one (20 versus 15), at least in the nucleotide context in which the motifs were tested in both studies. While the nucleotides immediately flanking a given motif can modulate frameshift level (19,20,36–38), they probably cannot turn an inefficient motif into a highly efficient one or vice versa, as suggested by one analysis in *E. coli* [see Table 3 in (36)]. That the above rules likely apply to other eukaryotic organisms is supported by studies on yeast and plant viruses (51,59–61). Several observations suggest that the *E. coli* rules are probably valid for many other bacterial species. A survey of the GtRNAdb database [(62); http://gtrnadb.ucsc.edu/] in June 2013 indicates that out of 431 different bacterial species, 168 possess only one type of lys-tRNA, with a _3′_UUU_5_′ anticodon, like *E. coli* (41). In these species, covering all the major bacterial phyla, the A_AA.R and V_VV.A_AA.R motifs should be as shift-prone as in *E. coli*. Interestingly, the same two types of motifs are highly prevalent (74.5%) among non-redundant IS elements from the IS*1* and IS*3* families present in the ISFinder database (Figure 5). Furthermore, among the 134 species present in the GtRNAdb database and in which IS*3* family transposable elements are found (ISFinder database, October 2012), 83 contain both types of lys-tRNA (_3′_UUU_5′_ and _3′_UUC_5′_ anticodons). ISs with an A_AA.G or V_VV.A_AA.G motif are present in 46 of these 83 species. Thus, presence of a lys-tRNA with a _3′_UUC_5′_ anticodon, which should pair perfectly with the AAG codon and thus reduce frameshifting (41), does not preclude the use of A_AA.G or V_VV.A_AA.G frameshift motifs in IS elements from many bacterial species.

A common feature of tetramer and heptamers motifs is the preferred identity for the Z nucleotide, A or U, constituting the first two bases of the ZZN codon. This suggests that a weak tRNA-codon pairing interaction in the A site is a universal pre-requisite for high level -1 frameshifting (17). The major differences concern the identity of the X and N nucleotides. While N_euk_ can be A, C or U, N_prok_ identity is linked to that of Z so that ZZN_prok_ must be all purines or all pyrimidines to achieve high frameshifting level. In terms of tRNA-codon relations, this suggests that the prokaryotic ribosome tolerates less readily a non-cognate interaction after frameshifting (e.g. following a shift from AAC to AAA) than its eukaryotic counterpart. One possibility is that the bacterial ribosome still monitors the correctness of the codon–anticodon pairing in the A site even after frameshifting. Concerning the ribosomal P site tRNA, which has to shift from XXZ to XXX, the prokaryotic ribosome still displays the same preference for cognate pairing in the new frame when Z is U. However, it is more eukaryotic-like when Z is A, a feature reflecting the high shiftiness of bacterial lys-tRNA_UUU_ especially when ZZN is AAG [Figures 3 and 4; (41)].

The previous paragraph highlighted the most efficient motifs and their properties, as revealed in three particular contexts (IS*911*, IS*3* and no-stimulator; see Figure 2). Overall, about 61% of the heptamers are significantly shift-prone, to very different extent, in the absence of stimulators, a feat confirming that the motif, i.e. tRNA re-pairing, is the primary determinant of -1 frameshifting. Stimulatory elements cannot induce frameshifting by themselves. They likely facilitate tRNA re-pairing by causing ribosome pausing (27–29) and by promoting mRNA realignment (30–32). It is interesting to note that heptamers of low efficiency in *E. coli* (Figure 4), like A_AA.A_AA.C and G_GG.A_AA.C, are nevertheless very likely used for programmed frameshifting by bacterial IS elements [Figure 5; (10)].

### Distribution of heptameric frameshift motifs in genes from 28 *E. coli* strains and 7 other enterobacterial strains

Study of the distribution of the 64 X_XXZ_ZZN heptamers in 22 703 sequences, selected to constitute our nrMEG, revealed that about 66% of the motifs were underrepresented to different extents (Figure 6, Supplementary Table S3). However, there was no significant anticorrelation between the observed frameshifting efficiency and the underrepresentation of shifty patterns when all the genes are taken into account or when only a subset of genes categorized as highly expressed was considered (Figure 7). The latter finding was unexpected, because it is believed that the deleterious effect of frameshift-prone patterns, at least in highly expressed genes (6), should increase with increased frameshifting efficiency and thus augment the pressure for selection against these sequences in protein coding regions. At this point we may only speculate about possible reasons. One reason could be the dependency of frameshifting on the context. Such context effects, involving nucleotides located immediately upstream or downstream of some motifs (tetramers and heptamers), were revealed through directed mutagenesis of frameshifting signals of prokaryotic and eukaryotic origin (19,20,36–38). Our experimental assays were carried out in a limited set of nucleotide context surrounding the patterns and, therefore, our results may not reflect the frameshifting efficiencies of these patterns in all their native contexts. Another possibility is that, even for the most efficient motifs placed in the best immediate context, frameshifting frequency remains sufficiently low in the absence of stimulatory elements, so as to have no detrimental effect on bacterial fitness. For the three best V_VV.A_AA.G heptamers(V = [C,A,G]) this frequency is at around 0.3% (Figure 4). From another study, we know that frameshifting on these motifs could be increased by about 12.4-fold at most, i.e. going up to 3.7%, by modifying the 3′ context (36). But this is still much lower if compared to the cumulative effect of background translational errors: missense errors and drop-off have a total estimated frequency of about 5×10^−4^ per amino-acid and thus would result in ∼18% of incorrect chains for a 500 amino-acids protein (63).

### Search for genes potentially using -1 frameshifting

Previous attempts to find novel recoded genes in bacteria used two different approaches, one based on search of frameshift-prone motifs (6,64), and the other based on the identification and characterization of disrupted coding sequences (11,13,14). The former led to identification of a few candidate genes only, but the search was restricted to a limited number of motifs and to one organism only, *E. coli*. The studies using the second approach were more exhaustive since they used all the available sequenced bacterial genomes. Consequently, they brought more candidates. A search of genes with disrupted open reading frames (ORFs) among 973 genomes initially revealed about 1000 candidate genes, 75% of which could be grouped into 64 clusters (11). Assuming an average number of 3130 protein-coding genes per genome, this gives a frequency of candidates of about 0.03%. Sequence comparison showed that 47 clusters contained genes from IS mobile genetic elements. Interestingly, a substantial proportion of them (22 clusters) may use programmed transcriptional realignment rather than translational -1 frameshifting (12 clusters) and in 9 clusters, both types of recoding may operate. The analysis with the GeneTack program of 1106 microbial genomes carried out by Antonov *et al.* (13,14) eventually revealed 4730 genes, potentially using frameshifting (in the +1 or -1 direction) or transcriptional realignment, which were grouped into 146 clusters. IS transposable elements genes are found in a minority of clusters, 17. Thus, other categories of genes of various functions predominate. However, if the absolute number of genes is considered, then IS elements prevail with a total number of 3317 genes. This probably reflects that ISs are prone to horizontal transfer and are often present in multiple copies in a genome. Assuming again an average number of 3130 protein-coding genes per genome, then the overall frequency of candidates found by Antonov *et al.* (14) among 1106 genomes is about 0.14%. One drawback of the disrupted-ORF approach is that it fails to detect cases where frameshifting would lead to a protein shorter than the product of normal translation. An example is provided by the *dnaX* gene family: while in *E.coli* and many bacteria frameshifting leads to a shorter protein, it results in a longer product in a more limited number of bacteria. The GeneTack analysis detected only the later cases in 17 genomes [see COF_239165634 in the GeneTack prokaryotic frameshift database; (14)].

In contrast, approaches primarily based on the search of frameshift motifs allow detection of both types of recoding outcomes, but the motifs are so short (e.g. 4 or 7 nucleotides for -1 frameshift motifs) that in the absence of proper filtering, too many candidates are found, even if presence of a potentially stimulatory structure downstream of the motif is an added condition. An illustration is provided by a study of the yeast genome: 20% of the ORFs (i.e. 1275 out of 6353) were found to contain one or more ‘strong’ -1 frameshift signal (7); by strong the authors mean that there is an efficient motif (as defined in the previous section) and a downstream structure (the total number of strong candidate frameshift regions was 1679). Since in 99% of the cases, -1 frameshifting leads to rapid premature termination, it was proposed, and then experimentally substantiated at least for a few genes (65), that -1 PRF is largely used in yeast for regulatory purpose rather than to generate, as is the case in IS elements or viruses, a fusion protein with a new carboxyl-terminal functional domain (7). A subsequent study, using a different method for structure prediction and scoring, lead to a much less optimistic evaluation: only 74 candidates of the former study were retained (66).

One aim of the present study was to identify genes containing 21 selected frameshift motifs within 36 individual genomes, 27 of which are from *E. coli* strains. The cumulated number of protein coding genes is 165 099 and among them 31 180 contain at least one of the 21 motifs, thus the overall frequency of motif-containing genes is 18.9%. After filtering and enrichment beyond the *E. coli* species, the outcome was a set of 69 clusters (i.e. a total 10 918 genes) each being a group of closely related genes where a given motif is conserved. Since about 10 392 019 genes were tested, the final yield of frameshift candidates was of 0.10%. This value is close to that obtained by Antonov *et al.* (14), therefore, suggesting achievement of a similar stringency by both searches. An internal validation of our method was provided by the fact that expected cases (*dnaX* gene, 7 IS elements and 2 bacteriophage genes; marked as **[true]** in Supplementary Tables S4 and S5 and in Figure 8) were found in the final set. Among the 59 remaining clusters, 1 is from IS elements, 3 are from prophages genes and 55 are from non-mobile cellular genes of known and unknown functions. In 57 of our 69 clusters, like in *E. coli dnaX* gene, but in contrast with ISs from the IS*1* and IS*3* families and all the 146 programmed frameshift clusters from the GeneTack database, frameshifting would lead to a product shorter than the protein resulting from normal translation (Figure 8B). Whether or not this type of frameshifting affects mRNA stability, as proposed for yeast candidates (7,65), remains to be determined. A majority of our clusters, 55, contain a conserved potential stimulatory element (as defined in Materials and Methods): there is an upstream SD in 2 clusters, a downstream hairpin in 44 and both types of stimulators in 9 (Figure 8C). However, assessment of the hairpins with the ΔΔG.nt^−1^ parameter suggested that they may be relevant in only 1 phage cluster and in 14 non-mobile genes clusters (Figure 9; Supplementary Table S8). Furthermore, if motif efficiency is taken as an additional constraint, only 8 clusters remain as best candidates for high level -1 frameshifting (marked with ** in Supplementary Table S8). As shown in Supplementary Table S7, there is a limited overlap between our 69 clusters and the 146 clusters of Antonov *et al.* (13,14), thus, demonstrating that the two approaches are complementary. The present study, in agreement with previous ones (11–14), suggests that recoding in bacteria is mostly found (at least in terms of absolute number of candidates genes) in IS transposable elements and in a few bacteriophage genes. However, if numbers of gene clusters are considered, then it appears that non-mobile genes clusters predominate. Information about the function of these genes, as found in the Ecogene database (67), is shown in Supplementary Table S5. Seventeen are of unknown function, four are predicted to be transcriptional regulators and the rest have different predicted functions.

Once candidates have been found, a critical issue, as stressed in a recent review (68), is their functional validation. This entails two steps: (i) the demonstration that there is frameshifting (or transcriptional slippage) on the predicted signal and (ii) the determination of the cellular function of the recoding product. Full functional analysis has not yet been carried out on the 146 GeneTack clusters or on the candidates genes reported in this study. Representatives of both studies, 20 of the GeneTack clusters (14) and 2 of our 8 best candidates (Supplementary Table S8), were tested for the first step only. Promisingly, 14 of the 20 GeneTack candidates and both of ours were found capable of eliciting frameshifting at different but substantial levels, i.e. from 0.3% to 63% [(14), Supplementary Figure S3]. The challenge is now to carry on with the complete experimental characterization of all candidates to establish which of them indeed use recoding to synthesize alternate proteins biologically pertinent for *E. coli* and other bacterial species.

## AVAILABILITY

Additional information about clusters is available at http://lapti.ucc.ie/heptameric_patterns_clusters/.

## SUPPLEMENTARY DATA

Supplementary Data are available at NAR Online.

SUPPLEMENTARY DATA
