# Supplementary material for: Analysis of tetra- and hepta-nucleotides motifs promoting -1 ribosomal frameshifting in Escherichia coli
Source: Nucleic Acids Res. 2014 May 28;42(11):7210–25. doi: 10.1093/nar/gku386 (PMC4066793; doi:10.1093/nar/gku386)
Supplement: SUPPLEMENTARY DATA [file supp_42_11_7210__index.html]

SUPPLEMENTARY DATA 

# Analysis of tetra- and hepta-nucleotides motifs promoting -1 ribosomal frameshifting in *Escherichia coli*

## SUPPLEMENTARY DATA

**Files in this Data Supplement:**

- Supplementary Data
