# Supplementary material for: Analysis of tetra- and hepta-nucleotides motifs promoting -1 ribosomal frameshifting in Escherichia coli
Source: Nucleic Acids Res. 2014 May 28;42(11):7210–25. doi: 10.1093/nar/gku386 (PMC4066793; doi:10.1093/nar/gku386)
Supplement: SUPPLEMENTARY DATA [file supp_gku386_nar-00572-v-2014-File011.pdf]

SUPPLEMENTARY DATA

Figure S1. Panel A: comparison of the relative frameshifting efficiencies of 44 X_XX.Z_ZZ.N heptamers tested in infectious bronchitis virus (IBV) eukaryotic context and IS*3* bacterial context. The frameshift frequency values of Brierley *et al.* (17) were used to generate panel A; for that, all values were normalized relative to that of the best motif, U_UU.A_AA.C (41.70%). Those for the IS*3* context (panel B) were normalized using the value for the C_CC.A_AA.G motif (54.4%; Figure 4). **Panel B**: Synopsis of the rules of -1 frameshifting on X_XX.Z_ZZ.N motifs in prokaryotes and eukaryotes as a function of the identity of the X, Z and N nucleotides. The "best" motifs category comprises those with a stimulatory efficiency of at least 0.2 times that of the most efficient as found in Figure 10 (*i.e.* C_CC.A_AA.G in the IS*3* prokaryotic context and U_UU.A_AA.C in the IBV eukaryotic context).

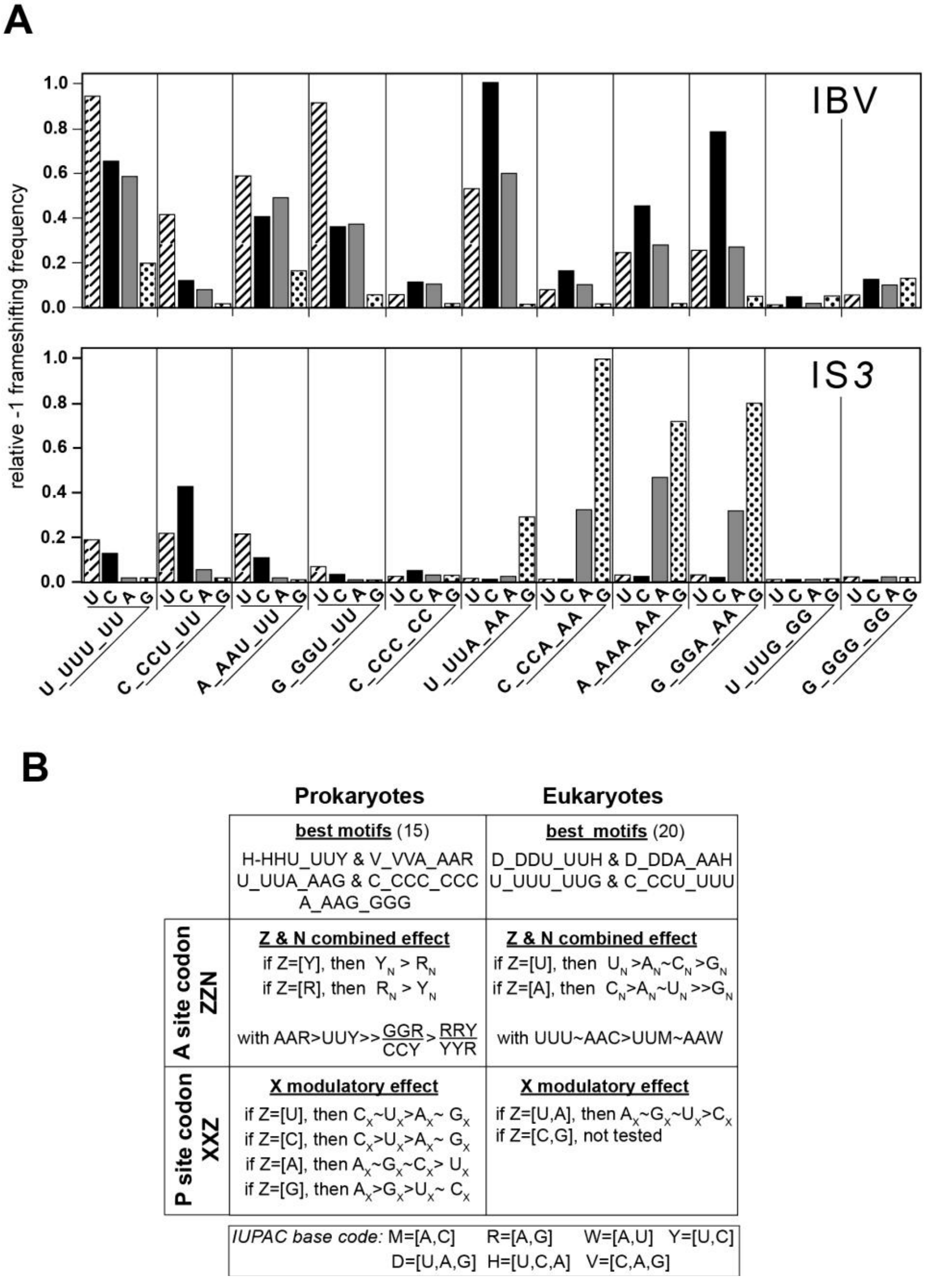

Figure S2. Analysis of potential frameshift stimulating hairpins in 271 members of the IS*3* family. Panel A shows the distribution of the ISs as a function the size of the spacer between the frameshift motif and the hairpin. Panel B displays the distribution of the same ISs as a function of the size of the first stem of the structure. Panel C is a plot of the value of $\Delta G_{unfold@37°C}.nt^{-1}$ as a function of the size of the structure.

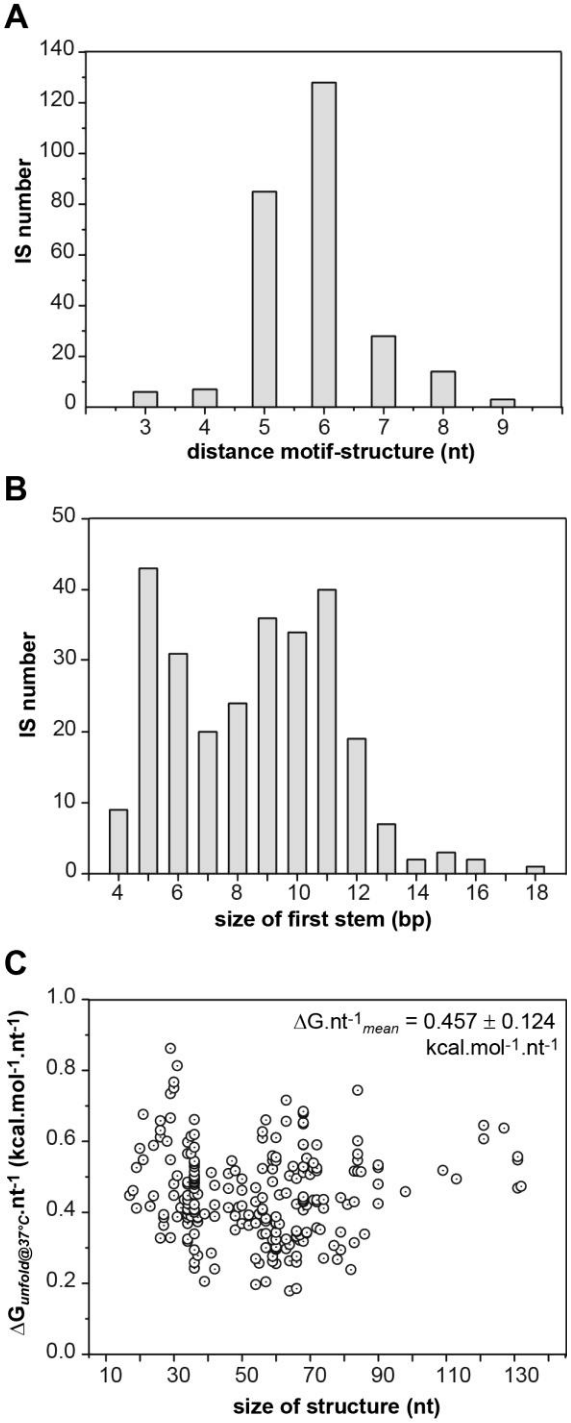

Figure S3. Determination of the -1 frameshifting capacity of two of the candidate clusters from this study. The A_AAG_GGG_15 (panel A) and T_TTA_AAG_11 (panel B) clusters were selected because they both contain a potential frameshift signal composed of an efficient motif associated with a downstream stimulator for the former (hairpin) or with an upstream stimulator for the latter (SD)(see also Table S8). The sequences shown in panels A and B were cloned into the pOFX310 reporter plasmid and their frameshifting frequencies were assessed by measuring LacZ activity as indicated in Materials and Methods. Both cloned regions have a moderate but significant frameshifting propensity since both are at a level well above the average background measured using the no-motif constructions (0.046 ± 0.002%).

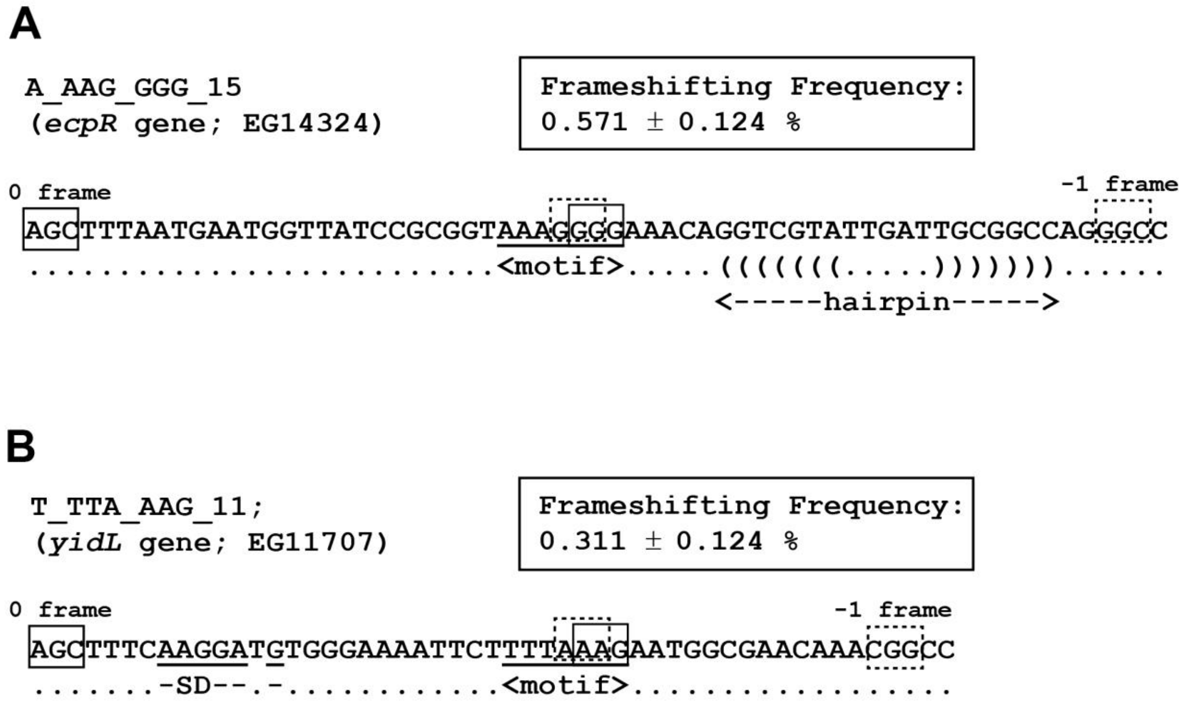

Table S1. List of the Refseq accession numbers and the respective organisms/strains that were used to create the integrated *E. coli* genome.

Refseq Accession	Organism
NC_000913	*Escherichia coli str. K-12 substr. MG1655*
NC_002655	*Escherichia coli O157:H7 str. EDL933*
NC_002695	*Escherichia coli O157:H7 str. Sakai*
NC_003197	*Salmonella enterica subsp. enterica serovar Typhimurium str. LT2*
NC_004337	*Shigella flexneri 2a str. 301*
NC_004431	*Escherichia coli CFT073*
NC_004741	*Shigella flexneri 2a str. 2457T*
NC_007384	*Shigella sonnei Ss046*
NC_007606	*Shigella dysenteriae Sd197*
NC_007613	*Shigella boydii Sb227*
NC_007946	*Escherichia coli UTI89*
NC_008253	*Escherichia coli 536*
NC_008258	*Shigella flexneri 5 str. 8401*
NC_008563	*Escherichia coli APEC O1*
NC_009800	*Escherichia coli HS*
NC_009801	*Escherichia coli E24377A*
NC_010468	*Escherichia coli ATCC 8739*
NC_010473	*Escherichia coli str. K-12 substr. DH10B*
NC_010498	*Escherichia coli SMS-3-5*
NC_010658	*Shigella boydii CDC 3083-94*
NC_011353	*Escherichia coli O157:H7 str. EC4115*
NC_011415	*Escherichia coli SE11*
NC_011601	*Escherichia coli O127:H6 str. E2348/69*

NC_011740	*Escherichia fergusonii ATCC 35469*
NC_011741	*Escherichia coli IAI1*
NC_011742	*Escherichia coli S88*
NC_011745	*Escherichia coli ED1a*
NC_011748	*Escherichia coli 55989*
NC_011750	*Escherichia coli IAI39*
NC_011751	*Escherichia coli UMN026*
NC_012759	*Escherichia coli BW2952*
NC_012947	*Escherichia coli BL21-Gold(DE3)pLysS AG'*
NC_012967	*Escherichia coli B str. REL606*
NC_013353	*Escherichia coli O103:H2 str. 12009*
NC_013361	*Escherichia coli O26:H11 str. 11368*
NC_013364	*Escherichia coli O111:H- str. 11128*
AC_000091	*Escherichia coli str. K-12 substr. W3110 strain K-12*

Table S2. -1 frameshifting efficiencies for the 16 Z_ZZN tetramers (upper panel) and their 16 mutated derivatives (lower panel) without or with a downstream stimulatory pseudoknot

motif	%FS-1 (without PK)	%FS-1 (with PK)
UUUU	0,139 ± 0.005	0,804 ± 0,059
UUUC	0,169 ± 0,011	1,784 ± 0,079
UUUA	0,150 ± 0,007	0,206 ± 0,024
UUUG	0,119 ± 0,020	0,138 ± 0,009
CCCU	0,164 ± 0,008	0,403 ± 0,050
CCCC	0,166 ± 0,008	0,406 ± 0,026
CCCA	0,153 ± 0,005	0,112 ± 0,013
CCCG	0,156 ± 0,008	0,168 ± 0,007
AAAU	0,139 ± 0,009	0,065 ± 0,011
AAAC	0,142 ± 0,008	0,065 ± 0,007
AAAA	0,125 ± 0,005	0,256 ± 0,007
AAAG	0,178 ± 0,006	5,640 ± 0,147
GGGU	0,126 ± 0,014	0,053 ± 0,009
GGGC	0,137 ± 0,014	0,047 ± 0,003
GGGA	0,155 ± 0,006	0,091 ± 0,009
GGGG	0,315 ± 0,012	0,218 ± 0,006

mutated motif	%FS-1 (without PK)	%FS-1 (with PK)
GUUU	0,099 ± 0,008	0,069 ± 0,005
GUUC	0,092 ± 0,003	0,066 ± 0,004
GUUA	0,117 ± 0,003	0,074 ± 0,008
GUUG	0,107 ± 0,010	0,071 ± 0,004
GCCU	0,119 ± 0,006	0,071 ± 0,006
GCCC	0,128 ± 0,005	0,072 ± 0,005
GCCA	0,120 ± 0,006	0,087 ± 0,007
GCCG	0,122 ± 0,005	0,081 ± 0,006
CAAU	0,121 ± 0,005	0,051 ± 0,004
CAAC	0,112 ± 0,005	0,044 ± 0,004
CAAA	0,100 ± 0,005	0,059 ± 0,005
CAAG	0,141 ± 0,006	0,929 ± 0,022
CGGU	0,121 ± 0,005	0,050 ± 0,001
CGGC	0,104 ± 0,005	0,040 ± 0,002
CGGA	0,184 ± 0,008	0,122 ± 0,002
CGGG	0,196 ± 0,006	0,105 ± 0,005

Table S3. Z-scores for the 3 phasings of the 64 XXXZZZN patterns in the nrMEG and experimentally determined -1 frameshifting efficiencies for the X_XXZ_ZZN phasing without stimulator or associated with the IS*911* or IS*3* stimulators.

A- XXXUUUN motifs

Motif	z-score			% -1 frameshifting (X_XXZ_ZZN)		
Phasing	X_XXZ_ZZN	XX_XZZ_ZN	XXX_ZZZ_N	no-Stim	IS*911*-Stim	IS*3*-Stim
UUUUUUU	-15.56	-13.84	-21.40	0.326±0.035	0.446±0.040	10.234±0.745
UUUUUUC	-1.10	-6.85	-16.27	0.121±0.006	0.893±0.089	7.581±0.674
UUUUUUA	7.23	0.15	-5.00	0.094±0.007	0.266±0.016	1.268±0.040
UUUUUUG	0.14	-9.55	-7.55	0.118±0.011	0.247±0.016	0.765±0.016
CCCUUUU	-3.55	-6.69	-5.47	0.212±0.005	2.995±0.353	11.653±0.597
CCCUUUC	0.12	3.51	-5.69	0.193±0.007	1.387±0.088	23.227±1.750
CCCUUUA	-3.23	-3.06	-0.99	0.365±0.030	0.678±0.031	2.896±0.128
CCCUUUG	-4.30	-4.60	-1.80	0.147±0.006	0.303±0.016	0.699±0.038
AAAUUUU	-9.10	-8.78	-10.92	0.070±0.002	0.355±0.005	11.441±1.068
AAAUUUC	-1.16	-7.53	-11.82	0.064±0.005	0.395±0.013	6.005±0.391
AAAUUUA	4.35	-2.79	-5.93	0.062±0.008	0.128±0.010	0.573±0.013
AAAUUUG	-0.08	-12.88	-5.42	0.068±0.005	0.181±0.014	0.381±0.011
GGGUUUU	-12.91	-2.20	-5.15	0.065±0.001	0.331±0.011	3.772±0.341
GGGUUUC	-16.56	1.55	-6.83	0.029±0.001	0.288±0.015	1.715±0.084
GGGUUUA	-12.37	8.55	1.93	0.042±0.002	0.133±0.005	0.468±0.029
GGGUUUG	-14.11	-5.52	2.74	0.065±0.005	0.123±0.005	0.256±0.009

B- XXXCCCN motifs

Motif	z-score			% -1 frameshifting		
Phasing	X_XXZ_ZZN	XX_XZZ_ZN	XXX_ZZZ_N	no-Stim	IS*911*-Stim	IS*3*-Stim
UUUCCCU	9.73	4.05	-0.63	0.119±0.015	0.127±0.008	0.684±0.012
UUUCCCC	12.17	4.90	0.85	0.125±0.006	0.185±0.007	0.977±0.109
UUUCCCA	-0.79	3.07	-0.86	0.104±0.005	0.180±0.008	0.627±0.014
UUUCCCG	14.48	20.05	-2.37	0.115±0.005	0.185±0.016	0.679±0.023
CCCCCCU	0.69	-2.41	1.47	0.101±0.004	0.204±0.014	1.727±0.076
CCCCCCC	0.48	-3.28	-2.53	0.144±0.013	0.286±0.030	4.314±0.128
CCCCCCA	-1.72	-4.26	0.72	0.123±0.009	0.321±0.017	1.825±0.044
CCCCCCG	-4.77	1.29	-0.29	0.121±0.013	0.363±0.018	1.587±0.107
AAACCCU	-4.16	-8.59	-0.26	0.057±0.003	0.080±0.002	0.718±0.027
AAACCCC	0.13	-8.43	-2.12	0.066±0.008	0.131±0.008	0.983±0.026
AAACCCA	-1.63	0.98	3.14	0.073±0.006	0.095±0.005	0.256±0.011
AAACCCG	1.93	12.24	2.70	0.057±0.004	0.114±0.013	0.363±0.011
GGGCCCU	-9.55	-12.72	-7.42	0.053±0.004	0.080±0.002	0.376±0.003
GGGCCCC	-7.53	-7.64	-6.87	0.048±0.002	0.089±0.002	0.418±0.025
GGGCCCA	-9.44	-11.00	-6.97	0.050±0.002	0.096±0.004	0.195±0.009
GGGCCCG	-16.49	-6.20	-9.62	0.051±0.002	0.073±0.003	0.218±0.011

C- XXXAAAN motifs

Motif	z-score			% -1 frameshifting		
Phasing	X_XXZ_ZZN	XX_XZZ_ZN	XXX_ZZZ_N	no-Stim	IS911-Stim	IS3-Stim
UUUAAAU	-2.13	XZZ= stop	4.73	0.131±0.002	0.074±0.004	0.275±0.010
UUUAAAC	-2.19	XZZ= stop	0.91	0.092±0.015	0.063±0.004	0.351±0.018
UUUAAAA	-4.61	XZZ= stop	-1.17	0.099±0.012	0.086±0.012	1.071±0.131
UUUAAAG	-2.74	XZZ= stop	8.55	0.177±0.009	0.354±0.014	15.837±2.365
CCCAAAU	-2.91	-6.86	-1.97	0.123±0.003	0.076±0.003	0.451±0.022
CCCAAAC	-5.92	-3.77	-0.78	0.179±0.006	0.149±0.005	0.488±0.013
CCCAAAA	-5.73	-6.65	1.21	0.192±0.007	0.442±0.027	16.943±4.534
CCCAAAG	-4.91	-1.45	-1.28	0.481±0.008	4.745±0.368	54.408±12.300
AAAAAAU	1.79	-3.59	-0.29	0.106±0.003	0.108±0.004	0.908±0.051
AAAAAAC	-6.74	-3.77	-1.09	0.163±0.010	0.189±0.017	0.739±0.044
AAAAAAA	-15.37	-28.34	-25.17	0.405±0.021	0.819±0.022	24.827±1.437
AAAAAAG	-10.10	-3.23	-0.65	0.291±0.015	4.255±0.282	39.004±4.011
GGGAAAU	6.85	3.96	4.54	0.069±0.017	0.112±0.003	1.054±0.008
GGGAAAC	-5.01	4.22	-5.74	0.164±0.004	0.222±0.006	0.888±0.025
GGGAAAA	5.62	7.69	10.03	0.117±0.011	0.511±0.013	16.983±1.114
GGGAAAG	-2.02	1.95	1.85	0.277±0.011	3.801±0.176	42.993±2.704

D- XXXGGGN motifs

Motif	z-score			% -1 frameshifting		
Phasing	X_XXZ_ZZN	XX_XZZ_ZN	XXX_ZZZ_N	no-Stim	IS911-Stim	IS3-Stim
UUUGGGU	-4.80	-8.13	-9.65	0.059±0.003	0.079±0.007	0.129±0.005
UUUGGGC	-7.25	-13.42	-12.27	0.087±0.003	0.073±0.002	0.100±0.004
UUUGGGA	1.88	-16.71	-11.71	0.040±0.002	0.082±0.002	0.166±0.015
UUUGGGG	6.40	-15.68	-14.68	0.064±0.004	0.107±0.006	0.181±0.004
CCCGGGU	-15.02	9.39	-4.53	0.053±0.003	0.044±0.002	0.110±0.003
CCCGGGC	-18.40	10.40	-3.43	0.043±0.005	0.029±0.001	0.107±0.002
CCCGGGA	-6.73	10.76	-4.63	0.039±0.006	0.050±0.004	0.243±0.001
CCCGGGG	-8.47	7.99	-8.05	0.053±0.003	0.055±0.003	0.412±0.014
AAAGGGU	-10.05	-2.51	8.03	0.134±0.003	0.368±0.015	1.598±0.058
AAAGGGC	-6.53	-1.89	10.02	0.171±0.003	0.587±0.0016	1.510±0.103
AAAGGGA	0.33	-0.39	9.78	0.136±0.004	0.666±0.007	3.474±0.159
AAAGGGG	1.08	-1.40	3.28	0.129±0.003	0.779±0.053	5.687±0.278
GGGGGGU	-14.97	-6.62	-6.70	0.018±0.002	0.131±0.006	0.875±0.047
GGGGGGC	-10.31	-0.22	-5.06	0.072±0.006	0.122±0.004	0.262±0.009
GGGGGGA	4.75	-6.38	-1.12	0.037±0.008	0.223±0.003	1.071±0.051
GGGGGGG	-5.18	-9.30	-11.11	0.030±0.002	0.088±0.009	1.073±0.037

Table S4. Clusters of candidate genes from transposable elements and prophages.

Cluster id[1]	number of genes[2]	RVSS /aln_{div}[3]	SD/ Structure[4]	Size of frameshift protein[5]	Gene name and Ecogene description[6]	
**Genes clusters from IS transposable elements**						
A_AAA_AAA_1	517	N/4.15	N/Y	L	*insM'*	Fam IS*3*/grpIS*3*/ IS*600* **[true];** transposase A
A_AAA_AAC_1	2303	Y/6.44	N/N	L	*insA4*	Fam IS*1*/ IS*1D* **[true];** transposase A
A_AAA_AAG_2	618	Y/6.51	N/Y	L	*insC*	Fam IS*3*/grpIS*2*/ IS*2* **[true];** transposase A
A_AAA_AAG_3	573	Y/8.74	Y/Y	L	*orfA*	Fam IS*3*/grpIS*3*/ IS*911* **[true]**; transposase A
A_AAA_AAG_4	604	Y/6.1	N/Y	L	*insC*	Fam IS*3*/grpIS*2*/ IS*2* **[true];** transposase A
A_AAA_AAG_5	59	N/0.02	Y/Y	L	*orfA*	Fam IS*3*/grpIS*150*/ IS*1397* **[true]**; transposase A
A_AAA_AAG_37	74	N/0.55	N/Y	L	*insJ*	Fam IS*3*/grpIS*150*/ IS*150* **[true];** transposase A
G_GGA_AAG_1	1609	N/02.89	N/Y	S	*insB1*	Fam IS*1*/ IS*1F*; transposase B
**Genes clusters from prophages**						
A_AAA_AAG_25	53	N/1.47	N/Y	L	ECs1801	tail assembly chaperone; **[true]**
A_AAG_GGA_2	91	N/0.72	N/Y	S	ECs1123	tail fiber protein
A_AAG_GGC_1	58	Y/0.84	N/N	S	ECs1759	prophage exonuclease
A_AAG_GGG_2	91	N/0.72	Y/N	S	ECs1123	tail fiber protein
G_GGA_AAG_4	74	N/1.14	N/Y	L	*gpG*	phage tail protein; **[true]**

Footnotes.

1- Each cluster is identified by its motif followed by a number. Two clusters are derived from the same gene sequences because these sequences contain 2 different motifs (A_AAG_GGA_2 and A_AAG_GGG_2) and two clusters come from closely related sequences (A_AAA_AAG_2 and A_AAA_AAG_4).

2- Number of genes in each cluster

3- RSSV stands for reduced variability at synonymous sites; Y (for yes) indicates that the p value is $\leq 10^{-4}$ (see Material and Methods). It means that the sequence around the motif is more conserved than expected, therefore suggesting selective pressure for local nucleotide sequence conservation. The aln_{div} parameter corresponds to an estimate of the mean number of phylogenetically independent nucleotide substitutions per alignment column.

4- Presence (Y) or absence (N) of potential frameshifting stimulatory elements flanking the motif, in the form of an upstream SD-like sequence or of a downstream structure.

5- S (for shorter) and L (for longer) respectively indicate that frameshifting leads to a product that is shorter or longer than the 0 frame product.

6- Clusters marked as **[true]** are those for which use of -1 PRF was either demonstrated (10,12) or can be safely inferred (*i.e.* A_AAA_AAG_5 or A_AAA_AAG_25); the description of each cluster is taken from the Ecogene 3.0 database (66).

Table S5. Clusters from non-mobile genes

Cluster id[1]	number of genes[2]	RVSS /aln_{div}[3]	SD/ Structure[4]	Size of frameshift protein[5]	Gene name and Ecogene description[6]
A_AAA_AAA_27	65	N/0.14	N/Y	S	*evgS*; sensor kinase for acid and drug resistance, cognate to EvgA
A_AAA_AAA_34	64	N/1.04	N/N	S	*yegX*; *p*redicted glycosyl hydrolase, family 25, function unknown
A_AAA_AAA_58	56	N/0.51	N/N	S	*yehI*; DUF4132 domain-containing protein, YehH paralog; function unknown
A_AAA_AAA_59	329	N/0.22	N/N	S	*fimC*; periplasmic chaperone for type 1 fimbriae; FimCD chaperone-usher transport
A_AAA_AAA_60	52	N/0.83	Y/Y	S	*yghT*; predicted ATP-binding protein, function unknown
A_AAA_AAA_64	54	N/0.89	N/N	S	*yihL*; predicted DNA-binding transcriptional regulator
A_AAA_AAA_84	38	N/0.05	N/Y	S	*yecT*; possible secreted protein or lipoprotein, function unknown
A_AAA_AAC_42	64	N/0.48	N/Y	S	*ygcR*; cell death gene, predicted electron transfer flavoprotein, beta subunit; function unknown
A_AAA_AAC_51	61	N/0.68	N/Y	S	*yidL*; predicted transcriptional regulator, AraC family; function unknown
A_AAA_AAC_55	127	N/2.70	N/Y	S	*cadB*; lysine-cadaverine antiporter
A_AAA_AAC_63	44	N/0.15	N/N	S	*yhiJ*; DUF4049 family protein, function unknown
A_AAA_AAG_6	198	Y/7.81	Y/Y	S	*dnaX* **[true];** DNA polymerase III holoenzyme, tau and gamma ATPase subunits
A_AAA_AAG_12	64	N/0.46	N/N	S	*gadW*; transcriptional activator of *gadA* and *gadBC;* repressor of *gadX;* AraC family
A_AAA_AAG_36	63	N/0.10	N/Y	S	*tdcR*; threonine dehydratase operon activator protein
A_AAA_AAG_38	46	N/0.52	Y/Y	L	*yrhB*; stable heat shock chaperone; predicted immunity protein; the sequence CCCTGA located 15nt after the motif was shown to promote +1 frameshifting (6)
A_AAG_GGA_9	80	N/1.46	N/Y	S	*pliG*; inhibitor of g-type lysozyme, periplasmic
A_AAG_GGG_15	63	N/0.40	N/Y	S	*ecpR*; putative transcriptional regulator for the ecp operon (*E. coli* common pilus); LuxR family
A_AAG_GGG_20	63	N/0.10	N/N	S	*tdcR*; see A_AAA_AAG_36
A_AAG_GGT_15	49	N/1.23	N/Y	S	*yaiS*; predicted PIG-L family deacetylase

C_CCT_TTA_12	48	N/0.78	Y/Y	S	*hcaR*; transcriptional activator for the *hca* (hydrocinnamic acid) operon; LysR family
C_CCT_TTC_14	56	N/0.34	N/Y	S	*pqqL*; predicted periplasmic zinc metalloendopeptidase, function unknown
C_CCT_TTC_18	91	N/1.00	N/Y	S	*cirA*; colicin IA outer membrane receptor and translocator; ferric iron-catecholate transporter
C_CCT_TTG_12	38	N/0.05	N/Y	L	*yecT*; see A_AAA_AAA_84
C_CCT_TTT_11	61	N/0.63	N/Y	S	*ybcH*; PRK09936 family protein; function unknown
C_CCT_TTT_16	60	N/0.90	N/N	S	*ynbD*; predicted phospholipid or protein phosphatase; predicted inner membrane protein
G_GGA_AAG_3	260	N/2.04	N/Y	S	*rhsD*; Rhs protein with DUF4329 family putative toxin domain; putative neighboring cell growth inhibitor
G_GGA_AAG_10	67	N/1.15	N/Y	S	*yjcZ*; mutational suppressor of *yhjH* motility defect; function unknown
T_TTA_AAG_6	65	N/0.14	N/Y	S	*evgS*; see A_AAA_AAA_27
T_TTA_AAG_9	64	N/0.62	N/Y	S	*yjcO*; Sel1 family TPR-like repeat protein; function unknown
T_TTA_AAG_11	61	N/0.68	Y/N	S	*yidL*; see A_AAA_AAC_51
T_TTA_AAG_15	13	N/0.42	N/Y	S	E2348C_3269; *E. coli* 0127:H6 hypothetical protein
T_TTA_AAT_10_A	79	N/0.70	N/Y	S	*kch*; voltage-gated potassium channel protein
T_TTA_AAT_10_B	79	N/0.70	N/Y	S	*kch*; see T_TTA_AAT_10_A
T_TTA_AAT_14	104	N/1.08	Y/Y	S	*hdeB*; periplasmic chaperone of acid-denatured proteins; RpoS regulon
T_TTA_AAT_15_A	65	N/0.14	N/N	S	*evgS*; see A_AAA_AAA_27
T_TTA_AAT_15_B	65	N/0.14	N/Y	S	*evgS*; see A_AAA_AAA_27
T_TTA_AAT_30	181	Y/1.83	N/N	S	*rpoS*; RNA polymerase subunit, stress and stationary phase sigma S; sigma 38
T_TTA_AAT_36	62	N/0.66	N/N	S	*yhbX*; putative EptAB family phosphoethanolamine transferase
T_TTA_AAT_46	68	N/1.65	N/Y	S	*ydjK*; putative sugar transporter, function unknown
T_TTA_AAT_49	56	N/0.52	N/Y	S	*yehI*; see A_AAA_AAA_58
T_TTA_AAT_56	47	N/0.64	N/N	S	*gfcC*; predicted periplasmic protein; required to make O-antigen capsule
T_TTA_AAT_58	53	N/0.92	N/Y	S	*yehM*; unknown function
T_TTC_CCA_20	66	N/0.58	N/N	S	*ydhT*; putative subunit of YdhYVW-XUT oxidoreductase complex
T_TTC_CCA_22	104	N/2.11	N/Y	S	*bcsE*; protein required for cellulose production

T_TTT_TTC_42	63	N/0.81	N/Y	S	*yegX*; see A_AAA_AAA_34
T_TTT_TTC_53	61	N/0.63	N/Y	S	*ybcH*; see C_CCT_TTT_11
T_TTT_TTC_57	53	N/0.34	N/N	S	*yddA*; putative ABC transporter permease/ATPase
T_TTT_TTC_60	82	N/0.67	Y/Y	S	*yjfK*; DUF2491 family protein
T_TTT_TTC_69	106	N/0.97	N/Y	S	*yfaA*; DUF2138 family protein, predicted host defense protein; function unknown
T_TTT_TTC_72	46	N/0.40	N/Y	S	*yliE*; membrane-anchored putative cyclic-di-GMP phosphodiesterase
T_TTT_TTC_74	48	N/0.76	N/Y	S	*dosP*; heme-regulated oxygen sensor, c-di-GMP phosphodiesterase
T_TTT_TTC_83	43	N/0.35	N/Y	S	*frvR*; putative frv operon regulator; contains a PTS EIIA domain
T_TTT_TTC_87	29	N/0.51	N/N	S	ECs4747; *E. coli* O157:H7 str. Sakai hypothetical protein
T_TTT_TTT_29	75	N/0.70	Y/Y	S	*fadK*; Acyl-CoA synthase, anaerobic
T_TTT_TTT_31	69	N/0.22	N/Y	L	*pyrL*; *pyrBI* operon regulatory leader peptide involved in regulation by attenuation; the motif is also part of a transcription terminator.
T_TTT_TTT_52	26	N/0.58	N/Y	S	*ymfA*; required for swarming phenotype, inner membrane protein, function unknown

[1,2,3,4,5 and 6] same footnotes as Table S4. 8 genes contain more than one motif, consequently there are several clusters for each: 4 clusters for *evgS* and 2 clusters for *kch*, *tdcR*, *ybcH*, *yecT*, *yegX*, *yehI* and *yidL*.

Table S6. Size of the proteins products for each cluster. One typical gene was selected and translated in frames 0 and -1 for each cluster. The size in amino acids of three products is indicated: (i) frame 0 product up to the last base of the X_XX.Z_ZZ.N motif (F0mot column), (ii) frameshift product (FSprot column), (iii) entire frame 0 product (F0tot column). The size of the FSprot and F0mot products relative to the corresponding F0tot protein is reported in the last two columns. Cluster ordering was carried out according to the relative size of the FSprot product. See Figure 8 (panels A& B) for a graphical presentation of these data.

Cluster order	Cluster id	F0mot (aa to motif)	FSprot (aa)	F0tot (aa)	FSprot/F0tot	F0mot/F0tot
1	T_TTT_TTC_74	15	18	799	0.023	0.019
2	C_CCT_TTC_18	7	20	663	0.030	0.011
3	T_TTA_AAT_30	12	12	330	0.036	0.036
4	T_TTT_TTC_72	28	30	782	0.038	0.036
5	T_TTT_TTC_60	5	13	219	0.059	0.023
6	T_TTA_AAT_10_A	30	35	417	0.084	0.072
7	T_TTA_AAT_15_A	88	116	1197	0.097	0.074
8	C_CCT_TTT_11	17	32	296	0.108	0.057
9	T_TTT_TTC_57	60	69	561	0.123	0.107
10	A_AAG_GGT_15	18	26	185	0.141	0.097
11	A_AAA_AAC_51	36	46	298	0.154	0.121
12	C_CCT_TTT_16	49	71	430	0.165	0.114
13	T_TTC_CCA_20	26	51	270	0.189	0.096
14	A_AAA_AAA_34	10	54	272	0.199	0.037
15	T_TTT_TTC_42	35	54	272	0.199	0.129
16	C_CCT_TTC_14	185	189	931	0.203	0.199
17	A_AAA_AAC_55	64	92	444	0.207	0.144
18	T_TTA_AAG_6	305	319	1197	0.266	0.255
19	A_AAA_AAC_42	57	70	259	0.270	0.220
20	A_AAA_AAG_36	21	32	114	0.281	0.184
21	A_AAG_GGG_20	22	32	114	0.281	0.193
22	T_TTA_AAG_9	28	65	229	0.284	0.122
23	T_TTA_AAT_15_B	342	358	1197	0.299	0.286
24	T_TTA_AAG_11	77	102	298	0.342	0.258
25	A_AAA_AAA_27	406	416	1197	0.348	0.339
26	A_AAG_GGC_1	339	341	823	0.414	0.412
27	T_TTA_AAG_15	103	126	274	0.460	0.376
28	G_GGA_AAG_3	626	674	1426	0.473	0.439
29	A_AAA_AAA_59	108	119	241	0.494	0.448
30	A_AAG_GGA_9	46	67	133	0.504	0.346
31	A_AAA_AAA_84	85	86	162	0.531	0.525
32	T_TTA_AAT_49	640	661	1210	0.546	0.529
33	A_AAG_GGG_15	87	108	196	0.551	0.444
34	A_AAA_AAA_60	76	131	230	0.570	0.330

35	C_CCT_TTA_12	174	176	296	0.595	0.588
36	A_AAG_GGG_2	259	272	439	0.620	0.590
37	A_AAA_AAG_6	430	431	643	0.670	0.669
38	T_TTT_TTC_53	184	200	296	0.676	0.622
39	T_TTA_AAT_14	73	73	108	0.676	0.676
40	T_TTA_AAT_36	366	369	541	0.682	0.677
41	T_TTA_AAT_46	305	324	459	0.706	0.664
42	A_AAA_AAG_12	172	175	242	0.723	0.711
43	G_GGA_AAG_1	113	125	168	0.744	0.673
44	T_TTT_TTT_52	118	118	153	0.771	0.771
45	T_TTT_TTC_83	445	458	582	0.787	0.765
46	T_TTT_TTC_69	445	461	562	0.820	0.792
47	A_AAA_AAA_58	982	995	1210	0.822	0.812
48	A_AAG_GGA_2	325	375	439	0.854	0.740
49	G_GGA_AAG_10	245	250	292	0.856	0.839
50	A_AAA_AAA_64	179	206	236	0.873	0.758
51	T_TTC_CCA_22	448	462	523	0.883	0.857
52	T_TTA_AAT_58	679	682	759	0.899	0.895
53	T_TTA_AAT_10_B	378	379	417	0.909	0.906
54	T_TTT_TTT_29	513	513	548	0.936	0.936
55	T_TTA_AAT_56	235	235	248	0.948	0.948
56	T_TTT_TTC_87	269	272	282	0.965	0.954
57	A_AAA_AAC_63	524	532	540	0.985	0.970
58	A_AAA_AAG_38	88	97	94	1.032	0.936
59	C_CCT_TTG_12	159	176	162	1.086	0.981
60	T_TTT_TTT_31	38	48	44	1.091	0.864
61	A_AAA_AAG_25	124	226	124	1.823	1.000
62	G_GGA_AAG_4	135	279	141	1.979	0.957
63	A_AAA_AAG_5	160	449	190	2.363	0.842
64	A_AAA_AAC_1	86	232	91	2.549	0.945
65	A_AAA_AAG_37	163	456	173	2.636	0.942
66	A_AAA_AAG_2	108	409	121	3.380	0.893
67	A_AAA_AAG_4	108	409	121	3.380	0.893
68	A_AAA_AAG_3	86	382	100	3.820	0.860
69	A_AAA_AAA_1	86	385	100	3.850	0.860

Table S7. Overlap between the 69 clusters from this study and the 146 programmed frameshift clusters from the GeneTack database (14).

**Cluster id** (this sudy)	**Genetack cluster number**[1]	**Gene name and comments**	
**Genes clusters from IS transposable elements**			
A_AAA_AAA_1	#1	*insM'*	Fam IS*3*/grpIS*3*/ IS*600* **[true]**
A_AAA_AAC_1	#5	*insA4*	Fam IS*1*/ IS*1D* **[true]**
A_AAA_AAG_2	#1	*insC*	Fam IS*3*/grpIS*2*/ IS*2* **[true]**
A_AAA_AAG_3	#1	*orfA*	Fam IS*3*/grpIS*3*/ IS*911* **[true]**
A_AAA_AAG_37	#1	*insJ*	Fam IS*3*/grpIS*150*/ IS*150* **[true]**
A_AAA_AAG_4	#1	*insC*	Fam IS*3*/grpIS*2*/ IS*2* **[true]**
A_AAA_AAG_5	#1	*orfA*	Fam IS*3*/grpIS*150*/ IS*1397* **[true]**
**Genes clusters from prophages**			
A_AAA_AAG_25	#10	ECs1801	tail assembly chaperone; **[true]**
**Non-mobile clusters**			
A_AAA_AAG_6	#20	*dnaX*	*dnaX*, **[true]**

[1] cluster numbers are from the table of programmed frameshift clusters available at http://topaz.gatech.edu/GeneTack/cgi/cof_signals.cgi?subset=bio_candidates

Table S8. Search of stimulatory elements upstream and downstream of the frameshift motif among the 69 clusters.

Cluster	SD[a]	HP[b]	SD & HP[a,b]	HP size (nt)	SP[c] (nt)	st1[d] (bp)	$\Delta G_{hp}.nt^{-1}$ [f] ± sd_{hp} [e]	$\Delta G_{av}.nt^{-1}$ ± sd_{hp} [f]	$\Delta\Delta G.nt^{-1}$ ± $sd_{\Delta\Delta}$ [g]	PRF -1[h]
**IS genes**										
A_AAA_AAA_1		0.85		34	6	9	0.380 ±0.011	0.120 ±0.074	0.260 ±0.040	T**
A_AAA_AAC_1		0.75		107	8	8	0.387 ±0.009	0.339 ±0.028	0.048 ±0.011	T
A_AAA_AAG_2		0.74		72	6	9	0.539 ±0.022	0.328 ±0.051	0.211 ±0.045	T**
A_AAA_AAG_3	0.68	0.62	0.44	57	6	7	0.352 ±0.074	0.228 ±0.044	0.123 ±0.067	T***
A_AAA_AAG_4		0.76		72	6	9	0.540 ±0.019	0.328 ±0.051	0.212 ±0.044	T**
A_AAA_AAG_5	1.00	1.00	1.00	38	3	4	0.435 ±0.009	0.203 ±0.077	0.232 ±0.019	T**
A_AAA_AAG_37		0.95		48	6	10	0.426 ±0.015	0.169 ±0.054	0.256 ±0.028	T**
G_GGA_AAG_1		0.93		59	3	4	0.263 ±0.015	0.184 ±0.046	0.079 ±0.025	
**Phage genes**										
A_AAA_AAG_25		0.85		130	5	7	0.415 ±0.021	0.395 ±0.023	0.020 ±0.023	T
A_AAG_GGA_2		0.86		18	4	4	0.480 ±0.050	0.177 ±0.131	0.303 ±0.107	
A_AAG_GGC_1										
A_AAG_GGG_2	1.00									**
G_GGA_AAG_4		0.51		54	7	4	0.302 ±0.022	0.282 ±0.112	0.020 ±0.095	T
**non-mobile genes**										
A_AAA_AAA_27		0.50		87	9	4	0.228 ±0.024	0.220 ±0.024	0.008 ±0.024	
A_AAA_AAA_34										
A_AAA_AAA_58										
A_AAA_AAA_59										
A_AAA_AAA_60	0.72	0.75	0.69	134	3	6	0.296 ±0.014	0.268 ±0.013	0.028 ±0.013	**
A_AAA_AAA_64										
A_AAA_AAA_84		1.00		91	9	4	0.211 ±0.029	0.204 ±0.020	0.007 ±0.023	
A_AAA_AAC_42		0.84		108	5	4	0.355 ±0.014	0.356 ±0.019	0.0001 ±0.017	
A_AAA_AAC_51		0.52		113	6	5	0.254 ±0.013	0.280 ±0.028	-0.026 ±0.026	
A_AAA_AAC_55		0.52		108	9	5	0.307 ±0.032	0.195 ±0.028	0.112 ±0.030	
A_AAA_AAC_63		0.82		121	6	4	0.231 ±0.000	0.256 ±0.035	-0.025 ±0.031	
A_AAA_AAG_6	0.68	0.71	0.56	30	6	5	0.663 ±0.057	0.270 ±0.132	0.393 ±0.118	T***

A_AAA_AAG_12										
A_AAA_AAG_36		1.00		103	6	6	0.127 ±0.021	0.118 ±0.018	0.008 ±0.020	
A_AAA_AAG_38	0.96	0.96	0.96	64	8	4	0.376 ±0.018	0.224 ±0.083	0.152 ±0.073	**
A_AAG_GGA_9		0.56		99	3	4	0.219 ±0.001	0.196 ±0.028	0.022 ±0.023	
A_AAG_GGG_15		0.98		19	5	7	0.558 ±0.000	0.054 ±0.078	0.504 ±0.062	**
A_AAG_GGG_20										
A_AAG_GGT_15		0.67		121	7	5	0.296 ±0.019	0.276 ±0.017	0.020 ±0.017	
C_CCT_TTA_12	0.60	0.94	0.60	98	3	4	0.324 ±0.009	0.305 ±0.027	0.019 ±0.021	
C_CCT_TTC_14		0.98		70	3	4	0.244 ±0.020	0.241 ±0.042	0.003 ±0.038	
C_CCT_TTC_18		0.68		38	8	10	0.310 ±0.011	0.152 ±0.055	0.158 ±0.044	
C_CCT_TTG_12		0.79		128	6	4	0.257 ±0.000	0.177 ±0.051	0.080 ±0.042	
C_CCT_TTT_11		0.90		95	6	4	0.279 ±0.020	0.235 ±0.025	0.044 ±0.025	
C_CCT_TTT_16										
G_GGA_AAG_3		0.59		81	5	4	0.392 ±0.024	0.162 ±0.093	0.231 ±0.079	**
G_GGA_AAG_10		0.84		54	7	6	0.350 ±0.003	0.217 ±0.105	0.132 ±0.090	
T_TTA_AAG_6		0.97		111	5	7	0.207 ±0.008	0.193 ±0.019	0.014 ±0.016	
T_TTA_AAG_9		0.92		54	9	7	0.316 ±0.014	0.277 ±0.086	0.039 ±0.065	
T_TTA_AAG_11	0.95									**
T_TTA_AAG_15		0.83		18	9	7	0.526 ±0.052	0.059 ±0.082	0.467 ±0.077	
T_TTA_AAT_10A		0.96		65	5	4	0.150 ±0.002	0.182 ±0.041	-0.032 ±0.035	
T_TTA_AAT_10B		0.96		99	8	4	0.199 ±0.011	0.213 ±0.051	-0.014 ±0.041	
T_TTA_AAT_14	0.96	0.88	0.82	132	9	6	0.162 ±0.006	0.237 ±0.062	-0.075 ±0.049	
T_TTA_AAT_15A										
T_TTA_AAT_15B		0.81		91	7	5	0.280 ±0.025	0.176 ±0.026	0.104 ±0.026	
T_TTA_AAT_30										
T_TTA_AAT_36										
T_TTA_AAT_46		0.78		106	5	6	0.272 ±0.016	0.237 ±0.027	0.034 ±0.025	
T_TTA_AAT_49		0.98		73	3	4	0.467 ±0.023	0.266 ±0.106	0.201 ±0.096	
T_TTA_AAT_56										
T_TTA_AAT_58		0.60		87	9	5	0.315 ±0.025	0.268 ±0.046	0.047 ±0.042	
T_TTC_CCA_20		0.89		112	4	4	0.266 ±0.030	0.266 ±0.019	0.0001 ±0.023	
T_TTC_CCA_22		0.62		87	7	4	0.356	0.321	0.035	

							±0.020	±0.055	±0.046	
T_TTT_TTC_42		0.92		112	6	4	0.153 ±0.014	0.290 ±0.069	-0.137 ±0.042	
T_TTT_TTC_53		0.95		85	8	8	0.338 ±0.011	0.295 ±0.037	0.043 ±0.029	
T_TTT_TTC_57										
T_TTT_TTC_60	0.96	0.69	0.69	109	3	5	0.303 ±0.009	0.300 ±0.025	0.004 ±0.021	**
T_TTT_TTC_69		0.90		50	3	4	0.221 ±0.006	0.125 ±0.041	0.095 ±0.032	
T_TTT_TTC_72		0.98		42	8	5	0.318 ±0.006	0.154 ±0.095	0.164 ±0.082	
T_TTT_TTC_74		0.77		88	4	4	0.269 ±0.022	0.229 ±0.024	0.040 ±0.023	
T_TTT_TTC_83		0.98		56	7	4	0.269 ±0.007	0.253 ±0.063	0.016 ±0.050	
T_TTT_TTC_87										
T_TTT_TTT_29	0.92	0.68	0.64	91	9	4	0.183 ±0.017	0.209 ±0.041	-0.026 ±0.033	**
T_TTT_TTT_31		0.97		97	3	4	0.216 ±0.001	0.251 ±0.037	-0.036 ±0.029	
T_TTT_TTT_52		0.88		96	4	4	0.234 ±0.019	0.140 ±0.033	0.094 ±0.029	

Footnotes.

[a] frequency of sequences with a Shine-Dalgarno-like sequence (SD) 6 to 17 nucleotides upstream of the frameshift motif.

[b] frequency of sequences with a hairpin structure (HP) starting 4 to 10 nucleotides downstream of the frameshift motif.

[c] SP corresponds to the number of nucleotides between the motif and the hairpin.

[d] st1 is the size in base-pairs of the first stem of the hairpin structure

[e] mean $\Delta G_{unfold@37°C}$ per nucleotide of the conserved hairpin (**$\Delta G_{hp}.nt^{-1}$**, $kcal.mol^{-1}.nt^{-1}$) and its standard deviation (**sd_{hp}**)

[f] average $\Delta G_{unfold@37°C}$ per nucleotide of structures predicted in a sliding window, of the same size as the corresponding conserved structure, moved over a 197 nt segment starting 4 nt after the motif (**$\Delta G_{av}.nt^{-1}$**, $kcal.mol^{-1}.nt^{-1}$) and its standard deviation (**sd_{av}**)

[g] $\Delta\Delta G.nt^{-1} = [\Delta G_{hp}.nt^{-1} - \Delta G_{av}.nt^{-1}]$ and its standard deviation (**$sd_{\Delta\Delta}$**)

[h] Clusters for which PRF-1 was demonstrated, or can be inferred, are indicated by T; ** or *** indicates the best PRF-1 candidates, *i.e.* clusters combining one of the best motif (as defined in Figure 11) and one (**) or two (***) potential stimulators (an upstream SD or an HP located 5 to 7 nt after the motif and for which $\Delta\Delta G.nt^{-1} \geq 0.09$).
